# A functional LSD1 coregulator screen reveals a novel transcriptional regulatory cascade connecting R-loop homeostasis with epigenetic regulation

**DOI:** 10.1093/nar/gkab180

**Published:** 2021-04-06

**Authors:** Sabine Pinter, Franziska Knodel, Michel Choudalakis, Philipp Schnee, Carolin Kroll, Marina Fuchs, Alexander Broehm, Sara Weirich, Mareike Roth, Stephan A Eisler, Johannes Zuber, Albert Jeltsch, Philipp Rathert

**Affiliations:** Department of Biochemistry, Institute of Biochemistry and Technical Biochemistry, University of Stuttgart, 70569 Stuttgart, Germany; Department of Biochemistry, Institute of Biochemistry and Technical Biochemistry, University of Stuttgart, 70569 Stuttgart, Germany; Department of Biochemistry, Institute of Biochemistry and Technical Biochemistry, University of Stuttgart, 70569 Stuttgart, Germany; Department of Biochemistry, Institute of Biochemistry and Technical Biochemistry, University of Stuttgart, 70569 Stuttgart, Germany; Department of Biochemistry, Institute of Biochemistry and Technical Biochemistry, University of Stuttgart, 70569 Stuttgart, Germany; Department of Biochemistry, Institute of Biochemistry and Technical Biochemistry, University of Stuttgart, 70569 Stuttgart, Germany; Department of Biochemistry, Institute of Biochemistry and Technical Biochemistry, University of Stuttgart, 70569 Stuttgart, Germany; Department of Biochemistry, Institute of Biochemistry and Technical Biochemistry, University of Stuttgart, 70569 Stuttgart, Germany; Research Institute of Molecular Pathology, Vienna BioCenter, Vienna, Austria; Stuttgart Research Center Systems Biology (SRCSB), University of Stuttgart, 70569 Stuttgart, Germany; Research Institute of Molecular Pathology, Vienna BioCenter, Vienna, Austria; Medical University of Vienna, Vienna BioCenter (VBC), Vienna, Austria; Department of Biochemistry, Institute of Biochemistry and Technical Biochemistry, University of Stuttgart, 70569 Stuttgart, Germany; Department of Biochemistry, Institute of Biochemistry and Technical Biochemistry, University of Stuttgart, 70569 Stuttgart, Germany

## Abstract

The lysine specific demethylase 1 (LSD1) plays a pivotal role in cellular differentiation by regulating the expression of key developmental genes in concert with different coregulatory proteins. This process is impaired in different cancer types and incompletely understood. To comprehensively identify functional coregulators of LSD1, we established a novel tractable fluorescent reporter system to monitor LSD1 activity in living cells. Combining this reporter system with a state-of-the-art multiplexed RNAi screen, we identify the DEAD-box helicase 19A (DDX19A) as a novel coregulator and demonstrate that suppression of *Ddx19a* results in an increase of R-loops and reduced LSD1-mediated gene silencing. We further show that DDX19A binds to tri-methylated lysine 27 of histone 3 (H3K27me3) and it regulates gene expression through the removal of transcription promoting R-loops. Our results uncover a novel transcriptional regulatory cascade where the downregulation of genes is dependent on the LSD1 mediated demethylation of histone H3 lysine 4 (H3K4). This allows the polycomb repressive complex 2 (PRC2) to methylate H3K27, which serves as a binding site for DDX19A. Finally, the binding of DDX19A leads to the efficient removal of R-loops at active promoters, which further de-represses LSD1 and PRC2, establishing a positive feedback loop leading to a robust repression of the target gene.

## Graphical Abstract

**Graphical Abstract:**
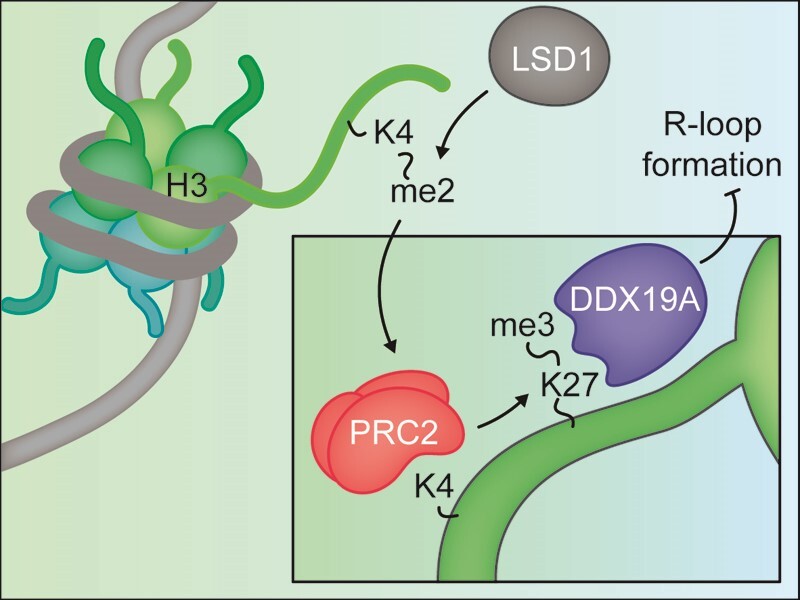
Model of the regulatory cascade for transcriptional repression, which depends on the removal of transcription-associated R-loops downstream of LSD1 activity.

## INTRODUCTION

The lysine specific demethylase 1 (LSD1, also known as KDM1A) has emerged as a critical regulator of essential physiological processes including the regulation of hormone receptor–mediated transcription (1), pluripotency and stem cell differentiation (2–5), cell cycle control (6) and DNA damage response (7). In agreement with the central role of LSD1 in such essential regulatory programs, LSD1 has been implicated in malignant transformation and maintenance of tumour pathogenesis in various ways. Overexpression of LSD1 has been observed in various tumour types (8–14) and imbalanced histone modifications, due to elevated LSD1 expression, are significantly associated with increased cellular growth and suppression of cell cycle regulatory proteins in a broad array of tissues. High levels of LSD1 have been shown to promote epithelial-to-mesenchymal transition (EMT) in breast cancer (BC) (15–17) and neuroblastoma (18), thereby contributing to cancer progression. Knockdown (KD) or inhibition of LSD1 reduces both the invasiveness and proliferative capacity of BC cells *in vitro* (19,20) and small molecules targeting LSD1 induce terminal differentiation of leukaemia cells (21,22). Thus, LSD1 represents a critical oncogene and potential therapeutic target in different cancer subtypes.

Most biological functions of LSD1 are associated with its activity to regulate the lysine methylation state of histones and non-histone proteins. LSD1 has been highlighted for its dual ability to stimulate or suppress gene expression (23–25) and was reported to demethylate lysine residues on histones as well as non-histone substrates such as p53 and DNMT1 (26,27). LSD1 mediates the demethylation of histone H3K4me1 and H3K4me2, thereby conducting a transcriptional repression (28–30), in part through downregulation of enhancer function (22). Contradictory to its corepressor function, LSD1 can directly activate the expression of target genes through demethylation of histone H3K9me2 (29–32). The exact molecular mechanism of its dual substrate specificity remains unclear, but recent publications support the hypothesis that a newly discovered alternative LSD1 splice variant (LSD1+8a) restricted to neuronal tissues is responsible for demethylation of H3K9 (33–35). LSD1 has been shown to be associated with actively transcribed genes in many cell types (22,23,28), which suggests that its H3K4 demethylation activity is blocked at these loci. In fact, the activity of LSD1 is tightly controlled and counterbalanced by associated coregulators and the interaction of LSD1 with coregulatory complexes, e.g. CoREST or the NuRD histone deacetylase (HDAC) transcription corepressor complexes, represents an important regulatory feature (1,32,36,37). Additionally, LSD1 activity was shown to be negatively regulated by the interaction with specific RNA structures (38), a feature also shown for other coregulator complexes, e.g. PRC2 (39,40). Finally, LSD1 can be subject to post-translational modifications (PTMs) which regulate its transcriptional activity (41).

This highlights the immense complexity of LSD1 regulation on different levels, which creates highly specific and tightly controlled LSD1 transcriptional outputs regulated by coordinated fine-tuning of the binding affinity of LSD1 to target loci and complex partners. Understanding the dependence of LSD1 function on accessory proteins will shed light on several signaling pathways and provide new therapeutic avenues by targeting factors that modulate LSD1 activity instead of or additionally to targeting LSD1 itself (42). Understanding how LSD1 evokes specific transcriptional profiles depending on its association with defined coregulators in distinct cellular contexts will be critical for the development of novel and more efficient LSD1-focused therapies. To date no comprehensive strategy to identify LSD1 coregulators and unravel their molecular function has been devised.

Recent methodologic advances introduced the chromatin *in vivo* assay (CiA) system, a variation of chemical induced proximity (CIP), as a novel method to investigate the consequences of locally induced alterations of the chromatin landscape after controlled recruitment of an epigenetic effector (43). CiA has successfully been applied to study the dynamics of heterochromatin formation at the Oct4 locus in mouse embryonic stem cells (mESCs) after the recruitment of HP1α (44), components of the PRC2 complex (45) and, combined with a high throughput small molecule library screen, to identify compounds inducing the formation of euchromatin (46). Additionally, CiA has been used to investigate the opposing effect of the BAF complex on PRC-induced heterochromatin formation, leading to the formation of accessible chromatin (47,48).

We aimed to identify and characterize functional coregulators that are required for LSD1 activity and adopted the CiA concept to generate a time-resolved fluorescent reporter system to monitor the activity of LSD1 in cells. To identify essential and novel coregulators of LSD1, we combined our fluorescent reporter system with a microRNA-embedded short hairpin RNA (shRNAmir) library focused on epigenetic effectors to perform a chromatin effector coregulator screen (ChECS). Our results provide a detailed functional view on the coregulator network of LSD1 in a multiplexed manner. Deeper characterization of one of the top hits from the screen, the DEAD-box helicase 19A (DDX19A) showed that RNA:DNA hybrid structures (also called R-loops) strongly interfered with the activity of LSD1. Our data reveal a novel regulatory cascade, which enables LSD1 induced transcriptional repression via a three-step mechanism. The decrease of H3K4 methylation at a particular genomic region induced by the activity of LSD1 leads to the recruitment of PRC2 to introduce H3K27 methylation. This modification serves as a signal for DDX19A, which binds to H3K27me3 via a yet unknown motif and removes R-loops. This de-represses LSD1 and PRC2 establishing a positive feedback loop leading to a strong repression of transcription at the targeted region.

## MATERIALS AND METHODS

### Plasmids

The fluorescent reporter expressing *mCherry* from a synthetic promoter (*synP*), consisting of six *tetO* binding sites upstream of an *EF1a* promoter, was cloned into pMSCV vector, on which expression of *mCherry* was driven by the *synP* promoter element and coupled to *Blasticidin* resistance *via* a *P2A* (pMSCV-tetO-EF1a-mCherry-2A-Blasti). The *rTetR-LSD1* fusion construct was cloned into a *pRRL* backbone by standard cloning methods. The LSD1 construct was kindly provided by Tim Somervaille. Expression was driven from an *SFFV* promoter and coupled to *Hygromycin* resistance via a *P2A* sequence (pRRL-rTetR-LSD1-P2A-Hygro). shRNA guides were cloned into the SGEN vector (49).

### Antibodies

Antibodies used for ChIP were H3K9me3 (ab8898, Abcam), H3K4me2 (ab7766, Abcam and #39141, Active Motif), H3K27me3 (#39155, Active Motif), H3K27Ac (ab4729, Abcam) and KDM1/LSD1 (ab17721, Abcam). Primary antibodies used for immunodetection after Western Blot were TetR monoclonal antibody 9G9 (#631131, TAKARA), DDX19A (orb242165, Biorbyt or ab108462, Abcam), RNA:DNA Hybrid Antibody, clone S9.6 (MABE1095, MERCK/Sigma-Aldrich) and KDM1A (#61607 and #39186, Active Motif). Secondary antibody used for immunofluorescence was the Goat Anti-Mouse IgG H&L (Alexa Fluor® 594, ab150116, Abcam). Antibody used for detection of DDX19A-GST was the goat anti-GST antibody (GE Healthcare, #27–4577-01). Secondary antibodies for analysis of Western Blots were either coupled to horseradish peroxidase (GE Healthcare) or to IRDye^®^ 800CW (ab216773, Abcam).

### Pooled RNAi screening

After spiking in control shRNAs at equimolar amounts, the shRNA-mirE library (5451 shRNAs and 8 control shRNAs) targeting 1010 chromatin-associated murine genes was transduced into NIH/3T3 cells expressing the *synP-mCherry* reporter and rTetR-LSD1. To ensure library representation, a total of 30 million cells were infected with 10% transduction efficiency using conditions that predominantly lead to a single retroviral integration and represent each shRNA in a calculated number of >500 cells. Cells were split into replicates and selected with 2.5 mg/ml Neomycin for 7 days before starting treatment with 1 μg/ml doxycycline. Throughout selection >3 × 10^6^ cells per replicate were maintained at each passage to preserve library representation. After 14 days of DOX treatment, cells were sorted into *mCherry*-positive (top 6–8%, minimum of 5 × 10^5^ cells) and *mCherry*-negative (lowest 75–80%, minimum of 6 × 10^6^ cells, see Supplementary Figure S2) populations using a FACS Aria III. Genomic DNA for both populations and 5 replicates was isolated with phenol-extraction using PhaseLock tubes, followed by ethanol precipitation. For each sample, DNA from at least 10^6^ cells was used as template in multiple parallel 50-μl PCR reactions, each containing 1 μg template, 1× AmpliTaq Gold buffer, 0.2 mM of each dNTP, 2 mM MgCl_2_, 0.3 μM of each primer and 1.25 U AmpliTaq Gold Polymerase (Life Technologies). In a first round of PCRs, random barcodes and sample barcodes were added to the shRNA sequences using the following cycling parameters: 95°C for 10 min; 28 cycles of (95°C for 30 s, 54°C for 45 s and 72°C for 60 s); 72°C for 7 min and primers *MM2P51_for* and *MM2P71_rev* (Supplementary Table S3). PCR products were combined for each sample, purified from a 1% agarose gel and 20 ng per sample were transferred to a second round of PCR, using similar cycling parameters as for PCR1, but with only 10 ng template per reaction, 6 cycles of amplification and primers *MM2P52_for* and *MM2P72_rev_N708* (Supplementary Table S3). In the second PCR, standard Illumina P7 adaptors and the Illumina N708 index were added to the sequences (total product length = 428 bp). All primers used for the library preparation are listed in Supplementary Table S2. The final libraries were cleaned up from a 1% agarose gel, pooled and analysed on an Illumina HiSeq 3000 deep sequencer (150 bp read length including the 22 nucleotides of the guide strand), using standard Illumina primers. Sequence processing was performed using a public Galaxy server (www.usegalaxy.eu). All primary screen data are provided in Supplementary Data Table S1. For each shRNA, the number of matching reads was normalized to the total number of library-specific reads per lane and imported into Microsoft Excel for further analysis. Completely depleted shRNAs (0 reads at T0) obtained a fold depletion value of 1 × 10^−3^. The average enrichment score for each individual shRNA was calculated by dividing the geometric mean of the normalized reads of the mCherry+ population by the respective normalized reads mCherry– populations (mCherry+/mCherry–) across five replicates. The gene score was derived by summarizing the average enrichment score of all shRNAs per gene. *P*-values are based on a Poisson distribution of each shRNA in each individual replicate followed by the combination of all *P*-values across all replicates using Fisher′s method (cumulative χ^2^). χ^2^ is a chi-squared distribution with 2*k* degrees of freedom, where *k* is the number of tests being combined. This fact was used to determine the *P*-value for χ^2^ followed by a Bonferroni correction for multiple comparison to obtain a *P*-value for each investigated gene in the library.

### Cell culture, retroviral transduction and flow cytometry

NIH/3T3, Lenti-X 293T and Platinum-E retroviral packaging cell lines were cultivated in DMEM high glucose media (Sigma-Aldrich) supplemented with 10% FBS, 20 mM glutamate, 10 mM sodium pyruvate, 10 mM HEPES (pH 7.3), 100 U/ml penicillin and 100 mg/ml streptomycin in an incubator providing 37°C and 5% CO_2_. For retroviral packaging of pMSCV vectors, 20 μg of plasmid were precipitated for 20 min in HBS buffer (140 mM NaCl, 25 mM HEPES, 0.75 mM Na_2_HPO_4_, pH 7.0) together with 125 mM CaCl_2_ and 10 μg GagPol helper plasmid. The mix was added to a 10 cm dish with Platinum-E cells growing at 75–85% confluence in supplemented DMEM. After 16 and 24 h, the media was replaced with fresh DMEM. Supernatant containing the virus was gathered 40–50 h after transfection, filtered through a 0.45 μm filter and added to the target cells at 50–70% confluence. Antibiotic selection for *pMSCV-tetO-EF1a-mCherry-2A-Blasti* with 10 μg/ml Blasticidin was started 2 days after transduction and kept up for 7 days. For retroviral packaging of pRRL-vectors, plasmids were mixed with helper plasmids *pCMVR8.74* (pCMVR8.74 was a gift from Didier Trono, Addgene #22036) and *pCAG-Eco* (pCAG-Eco was a gift from Arthur Nienhuis & Patrick Salmon, Addgene #35617) and 3× (w/w) excess of polyethyleneimine 25K in serum free DMEM. The mix was added to Lenti-X cells residing in supplemented DMEM at 75–90% confluence. Media exchanges and transduction of target cells was performed as described for pMSCV. Cells expressing *pRRL-rTetR-LSD1-P2A-Hygro* were selected with 500 μg/ml Hygromycin and cells expressing SGEN with 2.5 mg/ml Neomycin for 7 days. Recruitment of rTetR-LSD1 was started 12 days after transduction with SGEN by treatment with 1μg/ml Doxycycline. Inhibitor treatment with 10 μM GSK-LSD1, 5 μM GSK343 (Sigma-Aldrich) or 400 nM TSA (Sigma-Aldrich) was started in parallel with the first DOX induction and maintained for the indicated number of days. Expression of *GFP* and *mCherry* was analysed every 1–3 days using a MACSQuant Vyb flow cytometer.

### ChIP-qPCR

For H3K4me2-, H3K27me3- and H3K27ac-ChIP experiments, NIH/3T3 stably expressing the *synP-mCherry* reporter, rTetR-LSD1 wt or K661A and if indicated, the respective shRNA, were treated with 1 μg/ml doxycycline for 4 days. Cells were washed once with 1× PBS, before incubation with 1% formaldehyde in 1× PBS for 15 min at room temperature. Crosslinking was quenched with 225 mM glycine for 5 min. Cells were washed twice with 1× PBS and harvested with a Corning^®^ cell scraper in 10 ml 1× PBS per 10 × 10^6^ cells. Cells were centrifuged for 8 min at 600 × *g* and the pellet was washed again with 10 ml 1× PBS, 500 nM TSA per 10 × 10^6^ cells. Pellets were split into aliquots of 5 × 10^6^ cells, snap frozen and stored at –80°C until use. For preparation of mononucleosomes, each pellet was lysed in 125 μl lysis buffer (10 mM Tris–HCl pH 7.4, 2 mM MgCl_2_, 0.6% Igepal-Nonidet P40, 0.5 mM PMSF, 1 mM DTT, cOmplete™ EDTA-free PIC, 5 mM sodium-butyrate) for 15 min on ice. Samples were digested with 300 U micrococcal nuclease for 16 min at 37°C. The reaction was put on ice and stopped by addition of 8 μM EDTA, 0.1% Triton X-100 and 0.1% sodium deoxycholate. Samples were diluted by addition of 800 μl Complete IP buffer (20 mM Tris–HCl pH 8.0, 2 mM EDTA, 150 mM NaCl, 0.1% Triton X-100, 1 mM PMSF, cOmplete™ EDTA-free PIC, 5 mM sodium-butyrate) and clarified by centrifugation at 15 000 × *g* for 10 min at 4°C. The supernatant was split into aliquots of 40–70 μg chromatin and snap frozen. Before IP, *Drosophila melanogaster* mononucleosomes were added to the NIH/3T3 chromatin samples as spike-in control (2–3.5 μg = 5% of total chromatin). Ten percent of the sample was taken as input. For pre-clearing, 2.5 μg of rabbit/mouse IgG (depending on the species of antibody used for IP) and 10 μl of Dynabeads^®^ Protein G were incubated with the sample for 2 h at 4°C with constant rotation. The beads were removed using a magnetic rack and the sample was split into halves for IP/IgG control. 2.5 μg of ChIP antibody or IgG were added to the samples and incubated over night at 4°C with constant rotation. 20 μl Dynabeads^®^ Protein G per sample were blocked overnight in Complete IP buffer with 0.1 mg/ml BSA. Pre-blocked beads were incubated with the samples for 2 h at 4°C with rotation to bind antibodies. Beads were washed twice with low salt buffer (20 mM Tris–HCl pH 8.0, 2 mM EDTA, 150 mM NaCl, 1% Triton X-100, 0.1% SDS), twice with high salt buffer (20 mM Tris–HCl pH 8.0, 2 mM EDTA, 500 mM NaCl, 1% Triton X-100, 0.1% SDS) and once with TE buffer (10 mM Tris–HCl pH 8.0, 1 mM EDTA) to remove unspecific binding. Bound chromatin was eluted from the beads by resuspending in 100 μl SDS elution buffer (1% SDS, 100 mM NaHCO_3_) and rotating for 30 min at room temperature. Elution was performed twice and the eluates were combined. Samples were de-crosslinked for 16 h at 65°C with 2 μg RNase A and 270 mM NaCl. Proteinase digest was performed for 2 h at 45°C with 60 μg Proteinase K. DNA fragments were extracted using the ChIP DNA Purification Kit (Active Motif), and amplified using ORA™SEE qPCR reagent (HighQ) and qPCR primers amplifying a 120 bp fragment of the *EF1a* promoter. Cq values were normalized to input and *Drosophila* spike-in control. ChIP for H3K9me3 was performed the same way, except that the samples were harvested after 14 days of DOX treatment and not crosslinked before fragmentation and IP.

### ChIP-seq

LSD1 ChIP-seq was performed using the ChIP-IT High Sensitivity® Kit (Active Motif) following manufacturer’s instructions. In brief, 2 × 10^7^ NIH/3T3 cells were fixed and harvested as described in the protocol. Fragmentation was performed in aliquots of 5 × 10^6^ cells using an EpiShear Probe Sonicator (Active Motif) for 39 cycles (20 s pulse, 30 s pause, 40% amplitude), aliquots were united again and 5% were taken for input. To increase amount of precipitated chromatin, 3 × 25 μg chromatin were used for three independent IPs with 4 μg LSD1 antibody (ab17721, Abcam) each, following manufacturer's protocol. During DNA clean-up, the three samples were loaded onto two columns and the final eluates were united. Library preparation was performed using the NEBNext^®^ Ultra™ II DNA Library Prep Kit following manufacturer′s protocol. 1 μg of input DNA and 50% of precipitated ChIP DNA were used. After end repair and adapter ligation, the input was amplified using standard Illumina primers i705 and i503 for 3 cycles, the ChIP sample was amplified using i706 + i504 for 11 cycles. Libraries were analysed on a LabChip^®^ GX Touch™ Nucleic Acid Analyzer. Fragments with a size of 250–700 bp were sequenced on an Illumina HiSeq3000 using standard Illumina protocols.

### ChIP-Seq data analysis

Data analysis was performed on a public Galaxy server (www.usegalaxy.eu). After quality control, the remaining reads were aligned to the respective genome (mouse: mm9, human: hg19) using Bowtie2. Reads with same start and end position on the same strand were removed from the alignment. To identify ChIP-seq peaks, we used the MACS2 peak finding algorithm (50). A threefold enrichment relative to input control samples was used for peak calling as well as the option to call broad peaks. Building a shifting model was disabled and the small nearby and large nearby region parameters were set to 5000 and 20 000, respectively. The extension size was set to the respective median insert size of the ChIP-seq treatment sample for paired-end data and the estimated fragment size for single-end data. Downstream analysis was performed using the deepTools2 (51) suite using the multiBigwigSummary function to compute the average scores for each of the bigWig files in every genomic region. This analysis was performed for the entire genome by running the program in bins mode. Subsequently the result was plotted using the plotPCA and plotCorrelation functions. Peaks were assigned to the respective genes using ChIP-enrich (52) by assigning peaks to the closest upstream/downstream TSS.

The NIH/3T3 LSD1 ChIP-seq data of our study is available at Gene Expression Omnibus (https://www.ncbi.nlm.nih.gov/geo/) entry GSE158441.

The following ChIP-seq tracks were obtained from published data sets in K562 cells and mapped to hg19: H3K27ac (Encode sample ENCFF384ZZM), H3K27me3 (Encode sample ENCFF936BVT), H3K9me3 (Encode sample ENCFF700FQH), H3K36me3 (Encode sample ENCFF223BKS), H3K4me1 (Encode sample ENCFF463AQS), H3K4me2 (Encode sample ENCFF778DNU), LSD1 (GEO sample GSM831002), R-ChIP (GEO sample GSM2551007/8), DRIP-seq (GEO sample GSM1720619), GQ-seq (GEO sample GSM2876090/1). K562 RNA-seq (GEO sample GSM1557077). The following ChIP-seq tracks were obtained from published data sets in NIH/3T3 cells and mapped to mm9: H3K9ac (GEO sample GSM1246687), H3K27me3 (GEO sample GSM1246690), H3K36me3 (GEO sample GSM1246692), H3K9me2 (GEO sample GSM1246688), H4ac (GEO sample GSM1418787), H3K4me3 (GEO sample GSM879920), DRIPc-seq (GEO sample GSM2104456), DRIP-seq (GEO sample GSM1720621). NIH/3T3 RNA-seq (Encode sample ENCFF001QSC).

### RNA:DNA hybrid IP (DRIP)

Based on a detailed assessment of various DRIP protocols (53), DRIP was performed as described, with slight adaptations to workflow #19 (53). In brief, NIH/3T3 cells were harvested, washed once with 1X PBS and cross-linked in 1% formaldehyde/PBS for 10 min at room temperature. Crosslinking was quenched with 500 mM glycine for 5 min at room temperature. Cells were lysed in 300 μl of ChIP lysis buffer (50mM HEPES–KOH at pH 7.5, 140 mM NaCl, 1 mM EDTA at pH 8, 1% Triton X-100, 0.1% sodium-deoxycholate, 1% SDS) per 2 million cells for 30 min on ice and homogenized with a syringe every 10 min. Chromatin was fragmented by sonication using an EpiShear Probe Sonicator (Active Motif) for 2 × 12 cycles (20 s pulse, 30 s pause, 40% amplitude). The fragmented chromatin was supplemented with 300 mM NaCl and 50 μg of RNase A in 450 μl TE buffer (10 mM Tris–HCl pH 8, 10 mM EDTA pH 8) and incubated at 37°C for 1 h. The cross-linked *D. melanogaster* mononucleosomes were treated in parallel to obtain DNA for spike-in controls. To remove proteins and reverse the cross-links, the samples were treated with 15 μl of Proteinase K (20 mg/ml; Thermo Fisher Scientific) at 65°C for 16 h. Nucleic acids were extracted in two rounds of phenol extraction using PhaseLock tubes (Eppendorf), followed by ethanol precipitation at –20°C overnight. 15 μl Dynabeads^®^ Protein G per sample were blocked overnight in 1% BSA in 1× PBS. Nucleic acid precipitate was collected by centrifugation at 15 000 × *g* for 30 min, 4°C, the pellet was washed once with 70% ethanol and air-dried at 25–30°C. The pellet was resuspended in 5 mM Tris–HCl, pH 8.5 and concentration was determined by NanoDrop. To immobilize the S9.6 antibody, pre-blocked Dynabeads^®^ Protein G were resuspended in 1 ml IP buffer (50 mM HEPES/KOH at pH 7.5; 0.14 M NaCl; 5 mM EDTA; 1% Triton X-100; 0.1% sodium-deoxycholate) and incubated with 2 μg of S9.6 antibody per sample for 4 h, 4°C. of Fragmented nucleic acids (1.5 μg) and 50 ng of *Drosophila* spike-in were added to the antibody/bead complexes and IP was performed overnight at 4°C. For the RNAseH1 controls, 1.5 μg of nucleic acids were digested with 40 U RNAse H (NEB) at 37°C overnight. The enzyme was inactivated by incubation for 20 min at 65°C and the sample was taken as input for the IP in parallel with the untreated samples. Beads were washed once with 1 ml IP wash 1 buffer (20 mM Tris pH 8.0, 2 mM EDTA, 50 mM NaCl, 1% Triton X-100, 0.1% SDS), twice with 1 ml high salt buffer (20 mM Tris pH 8, 2 mM EDTA, 500 mM NaCl, 1% Triton X-100, 0.01% SDS), once with 1 ml IP wash buffer 2 (10 mM Tris pH 8, 1 mM EDTA, 0.25 M LiCl, 1% NP-40, 1% sodium-deoxycholate) and twice in TE buffer (pH 8). Nucleic acids were eluted in 50 μl elution buffer (50 mM Tris pH 8, 10 mM EDTA, 1% SDS) for 15 min at 65°C and further purified with the NucleoSpin Gel and PCR Clean-up kit (Macherey-Nagel), nucleic acids were eluted in 50 μl of elution buffer (5 mM Tris, pH 8.5). DNA fragments were amplified using ORA™SEE qPCR reagent (HighQ) and qPCR primers amplifying a 120 bp fragment of the *EF1a* promoter. Cq values were normalized to input and Drosophila spike-in control.

### S9.6 antibody IP

100 μl Dynabeads^®^ Protein G were pre-blocked with 0.5% BSA/PBS for 2 h at 4°C. 10 × 10^6^ non-crosslinked NIH/3T3 cells were harvested, washed once in 1× PBS and lysed in 1 ml Cell Lysis Buffer (85 mM KCl, 5 mM HEPES pH 8, 0.5% NP-40, cOmplete™ EDTA-free PIC) for 15 min on ice. Nuclei were collected by spinning 1 min at 15 000 × *g*, 4°C. The pellet was resuspended in 750 μl RSB buffer (10 mM Tris–HCl pH 7.5, 200 mM NaCl, 2.5 mM MgCl_2_, cOmplete™ EDTA-free PIC) supplemented with 0.2% sodium-deoxycholate, 0.1% SDS, 0.05% sodium-lauroyl-sarcosinate and 0.5% Triton X-100. Cells were sonicated for 4 min in an EpiShear Probe Sonicator (20 s pulse, 30 s pause, 40%). After taking 5% as input, samples were transferred to a 15 ml falcon tube and diluted 1:4 by addition of 2.3 ml RSB with 0.5% Triton X-100 (RSB+T). The samples were subjected to pre-clearing with 5 μg mouse IgG and 35 μl Dynabeads^®^ Protein G for 1 h at 4°C. Magnetic beads were locked and the supernatant was split into three Eppendorf tubes. For the S9.6 antibody-specificity control, 40 U of RNAse H (NEB) were added to one of the tubes and all samples were incubated for 10 min at 37°C before adding the IP antibodies. The samples were subjected to IP with 4 μg of either the S9.6 antibody or mouse IgG and 32 μl pre-blocked beads per sample. 10 ng RNase A was added to each tube before rotating at 4°C for 2.5 h. Beads were washed 4× with 500 μl RSB+T and 2× with RSB. With each buffer change, beads were transferred to fresh low-binding tubes to minimize leftover unspecific binding. Proteins were eluted in 40 μl of 2× SDS sample buffer (125 mM Tris–HCl pH 6.8, 5% SDS, 0.004% Bromophenol Blue, 10% β-mercaptoethanol, 100 mM DTT, 20% glycerol) for 10 min at 70°C. The supernatant was transferred to fresh tubes and boiled at 95°C for 10 min for denaturation, along with the input sample mixed with 2× sample buffer. SDS-PAGE and immunodetection of proteins were performed as described for immunodetection of proteins.

### Immunodetection of proteins after SDS-PAGE

For the analysis of protein levels, cells were harvested 13 days after transduction and antibiotic selection. Pellets were lysed in cell lysis buffer (Cell Signaling Technology^®^) for 30 min on ice. After 10 and 20 min of incubation, the lysate was sonicated with an EpiShear Probe Sonicator (Active Motif) for 2 cycles of 20 s to release nuclear protein. The lysate was centrifuged at 15 000 × *g* for 10 min, the supernatant was mixed with 2× SDS sample buffer (125 mM Tris–HCl pH 6.8, 5% SDS, 0.004% Bromophenol Blue, 10% β-mercaptoethanol, 100 mM DTT, 20% glycerol) and boiled at 95°C for 10 min. Proteins were resolved by SDS-PAGE on a 12% polyacrylamide gel. Proteins were transferred to an Immobilon-FL PVDF membrane at 300 mA for 90 min using a wet-tank blotting system (BioRad). Proteins were detected using a target specific primary antibody at manufacturer′s recommendations in combination with a species-specific HRP- or IRDye^®^ 800CW-coupled secondary antibody. Imaging was performed on a FusionFX detection system (VILBER) using SuperSignal™ West Femto Chemiluminescence substrate (ThermoFisher Odyssey^®^ CLx imaging system (LI-COR).

### Gene expression analysis

For analysis of mRNA expression levels of *Ddx19a*, cells were harvested 13 days after transduction with the specific shRNAs (Supplementary Table S3) and antibiotic selection. RNA was extracted using the *RNeasy Plus mini Kit* (QIAGEN). Reverse transcription and quantitative PCR were performed in one step using the Luna® Universal One-Step RT-qPCR Kit (NEB) and a CFX Real-Time PCR detection system (Bio-Rad). Beta-2-Microglobulin was used for normalization. qRT-PCR primers are described in Supplementary Table S5.

### Protein purification

For GST-tag purification of GST-DDX19A, *E.coli BL21(DE3)* cells were transformed with 50 ng of pGEX-DDX19A plasmid and plated on LB agar with 35 μg/ml Chloramphenicol and 50 μg/ml Kanamycin. Subsequently, 50 ml LB/Kanamycin were inoculated with one colony and the starter-culture was cultivated at 37°C, 150 rpm for 6 h. 500 ml LB/Kanamycin were inoculated with 6 ml of starter culture and cultivated at 37°C, 150 rpm until OD_600_ = 0.7. Expression of GST-DDX19A was induced by addition of 500 μM IPTG and overexpression was performed at 20°C, 150 rpm for 14 h. Cells were harvested at 5000 × *g* for 15 min, 4°C. Pellets were washed once in 30 ml STE buffer (100 mM NaCl, 10 mM Tris–HCl pH 8, 1mM EDTA) and frozen at –20°C until use. For purification, pellets were resuspended in 30 ml sonication buffer (20 mM HEPES pH 7.5, 0.2 mM DTT, 500 mM KCl, 1 mM EDTA, 10% glycerol) with protease inhibitor and lysed by sonication using an EpiShear Probe Sonicator (Active Motif). The lysate was cleared by centrifugation and filtration through a 0.45 μm CHROMAFIL GF/PET-45/25 filter (MACHEREY-Nagel). Affinity chromatography was performed using an NGC™ Chromatography system (BIO-Rad) and Protino^®^ Glutathione Agarose 4B beads (MACHEREY-Nagel). Proteins were eluted in elution buffer (20 mM HEPES pH 7.5, 500 mM KCl, 0.2 mM DTT, 1 mM EDTA, 10% glycerol, 40 mM glutathione) and subjected to dialysis into storage buffer (20 mM HEPES pH 7.5, 200 mM KCl, 0.2 mM DTT, 1 mM EDTA, 10% glycerol). Aliquots were snap-frozen and stored at –80°C. For storage at –20°C, proteins were transferred to a different storage buffer (20 mM HEPES pH 7.5, 200 mM KCl, 0.2 mM DTT, 1 mM EDTA, 60% glycerol) by another round of dialysis.

### RNA:DNA unwinding assay

RNA:DNA unwinding assay was performed as described (54). In brief: RNA:DNA hybrids were annealed *in vitro* in 5 mM Tris/HCl pH 8.5. The sequence of the top RNA strand was: 5′‐GAAGCUGGGACUUCCGGGAGGAGAGUGCAA‐3′, and the sequence of the bottom DNA strand was 5′-CGGGTTGTCAAGAATTTTAACGGCCATTTCTGTGTTGCACTCTCCTCCCGGAAGTCCCAGCTTCTGTGTTTGTGACAAACGCAAGCTCATGTAAGTGCTC‐3′. The annealed RNA:DNA hybrid has a 5′ ssDNA overhang and is labeled with Cy-5. Unwinding experiments were carried out at 30°C for 60 min in 30 mM Tris–HCl (pH 7.5), 50 mM NaCl, 5 mM MgCl_2_, 2 mM DTT, 0.01% NP‐40, 0.1 mg/ml BSA, 4 mM ATP, 1 nM Cy-5‐labeled RNA:DNA hybrid substrate, in the presence of 4.79 μM recombinant DDX19A. The reaction was stopped by the addition of SDS to a final concentration of 0.5% and 20 ng proteinase K (20 mg/ml). The reaction was afterward loaded onto a 10% non-denaturing polyacrylamide gel and analysed using a FusionFX detection system (VILBER).

### Celluspot and peptide arrays

Peptide arrays containing peptides with a length of 15 amino acids were synthesized by spotting on a cellulose membrane using an Autospot peptide array synthesizer (Intavis AG) and the SPOT synthesis method (55). MODified™ Histone Peptide Arrays (Active Motif) or synthesized peptide arrays were blocked overnight in blocking solution (5% milk powder, 1× PBS, 0.1% Tween20) at 4°C. Both arrays were washed three times for 5 min with 1× PBS/Tween-20 and pre-incubated for 10 min in interaction buffer (100 mM KCl, 20 mM HEPES pH 7.5, 1 mM EDTA pH 8, 10% glycerol). Binding of DDX19A-GST was performed by incubation of 50 nM protein with the pre-blocked array in interaction buffer for 1 h at room temperature. The array was washed three times for 10 min in 1× PBS/Tween20 and incubated with an anti-GST antibody (GE Healthcare, #27-4577-01) in 5% non-fat dried milk/1x PBS/Tween-20 for 1 h at room temperature. The array was washed again as described and incubated with an anti-goat-HRP antibody in 5% milk/1× PBS/Tween-20 for 1 h. After repeated washing, twice for 10 min in PBS/Tween-20 and once for 10 min in PBS, the array was imaged using a FusionFX detection system (VILBER) and SuperSignal™ West Femto Chemiluminescence substrate (ThermoFisher). Synthesized peptide sequences were: H3K4_ARTKQTARKSTGGKA; H3K9 RTKQTARKSTGGKAP; H3K27 LATKAARKSAPATGG; H3K36 APATGGVKKPHRYRP; H4K20 GGAKRHRKVLRDNIQ

### Immunofluorescence microscopy

NIH/3T3 cells were cultivated until 70–90% confluency on microscopy coverslips. Cells were washed three times for 5 min with 2 ml PBS^Ca2+ Mg2+^ (Sigma-Aldrich). Cells were fixed for 10 min at room temperature in 4% paraformaldehyde. Cells were washed as described and permeabilized with 0.2% ice-cold TritonX-100 in PBS for 5 min. Cells were blocked in 2 ml 5% non-fat dried milk in PBS^Ca2+ Mg2+^ for 1h at room temperature. Primary antibody binding was performed overnight at 4°C with a concentration of 4 μg/ml antibody in PBS^Ca2+ Mg2+^/5% non-fat dried milk powder. Secondary antibody binding was performed at room temperature for 2 h with a concentration of 0.5 μg/ml antibody in PBS^Ca2+ Mg2+^/5% milk powder. Cells were stained with 1μg/ml DAPI in PBS^Ca2+ Mg2+^ for 3 min, washed again with PBS and mounted on microscopy slides using Mowiol^®^ 4–88 (Sigma-Aldrich).

### Image acquisition and analysis

For the quantification of the S9.6 staining, samples were analysed on a Zeiss Axio Observer.Z1 microscope equipped with a Plan-Apochromat 63×/1.40 Oil DIC M27 objective and an AxioCam MRm camera. The following excitation and emission filters were used: Blue channel: excitation filter 335–383 nm, emission filter 420–470 nm; red channel: excitation filter 538–562 nm, emission filter 570–640 nm; green channel: excitation filter 450–490 nm, emission filter 500–550 nm. Z-stacks covering the whole nucleus were acquired applying an interval of 450 nm, and images were subjected to deconvolution using a constrained iterative algorithm and the ZENblue version 2.3 software (Zeiss), before generating maximum intensity projections. Quantitative image analysis was done with CellProfiler™ version 2.2 (56). Nuclei were identified via the DAPI staining.

### Co-immunoprecipitation of LSD1

NIH/3T3 were harvested by trypsinization. The pellet was resuspended in 2× pellet volume of nuclear lysis buffer B (50 mM Tris–HCl pH 7.4, 20% glycerol, 1.5 mM MgCl_2_, 420 mM NaCl, 1 mM Na_3_VO_4_, 25 mM NaF) supplemented with Protease inhibitor and incubated on ice for 15 min. Lysate was homogenized with 25 strokes of a douncer (0.01–0.03 mm) and incubated with rotation at 4°C for 30 min. Lysate was cleared by spinning down at 4°C, 16 000 × *g*, 30min and the supernatant was transferred to a new tube. 1× DP buffer (50 mM Tris–HCl pH 7.4, 5% glycerol, 1.5 mM MgCl_2_, 150 mM NaCl, 1 mM Na_3_VO_4_, 5 mM NaF) with 0.4% NP40 and protease inhibitors (1.8 ml buffer to 1ml lysate) was added and the sample was incubated on ice for 10 min before clearing through ultracentrifugation (30 min, 4°C/43 000 rpm/TI50.2). The supernatant was transferred to a fresh tube and 5% input were taken. The sample was incubated with 1μg anti-KDM1A antibody per 1mg protein in the lysate over night at 4°C. 1.5-fold of loading capacity of Dynabeads protein G were added for 2 h at 4°C with rotation. Beads were washed twice with DP/NP40 buffer, twice with DP buffer and twice with 150 mM NaCl. Proteins were eluted in 30 μl of 2× SDS sample buffer (125 mM Tris–HCl pH 6.8, 5% SDS, 0.004% Bromophenol Blue, 10% β-mercaptoethanol, 100 mM DTT, 20% glycerol) for 10 min at 70°C and subjected to SDS-PAGE and immunodetection as described for gene expression analysis.

### Equilibrium peptide binding experiments

Determination of *K*_D_ of DDX19A and H3K27 was conducted using H3.1 peptide labeled with FITC. The H3.1 peptide comprising residues 16–34 of the H3.1 tail was unmodified or trimethylated at K27. Binding was analysed using a Jasco FP-8300 spectrofluorometer with an automatic polarizer (FDP-837). Acquisitions were performed at 23°C, with excitation at 495.0 nm and emission measured at 520 nm. Slit width was set to 5 nm. 50 nM of peptide were dissolved in 0.5 ml of anisotropy buffer (20 mM HEPES pH 7.5, 100 mM KCl, 0.1 mM DTT, 10% v/v glycerol). DDX19A diluted in dialysis buffer (20 mM HEPES pH 7.5, 200 mM KCl, 1 mM Na2-EDTA, 60% v/v glycerol, 0.2 mM DTT) was added stepwise. Titrations were conducted in at least 3 technical replicates. Control experiments were conducted with dialysis buffer without protein and the fluorescence anisotropy values were corrected accordingly. For determination of the *K*_D_-values for H3.1, the data were fitted to a simple binding equilibrium:

$$\begin{equation*}{\rm Signal} = {\rm{BL }} + {{ F*}}\frac{{ {{{c}}_{{\rm{DDX}}19{\rm{A}}}}}}{{{{{c}}_{{\rm{DDX}}19{\rm{A}}}} + {{{K}}_{\rm D}}}}\end{equation*}$$

With *K*_D_ = equilibrium dissociation constant, *F* = signal factor and BL = baseline.

### Statistical analyses

Results are presented as means ± standard error of the mean [s.e.m.]. Matching sets of samples (treated vs control) were normalized to the average of all samples in this replicate. Values were scaled to the average of all untreated replicates set to average = 1. If not stated otherwise, statistical significance was calculated by one-tailed unpaired t-test on two experimental conditions with p≤0.05 considered statistically significant. Statistical significance levels are denoted as follows: ****P ≤ 0.0001; ***P ≤ 0.001; **P≤ 0.01; *P ≤ 0.05; n.s. = non-significant. No statistical methods were used to predetermine sample size.

## RESULTS

### Development of a novel fluorescent reporter system to investigate LSD1 and associated cofactors in living cells

**Figure 1.**
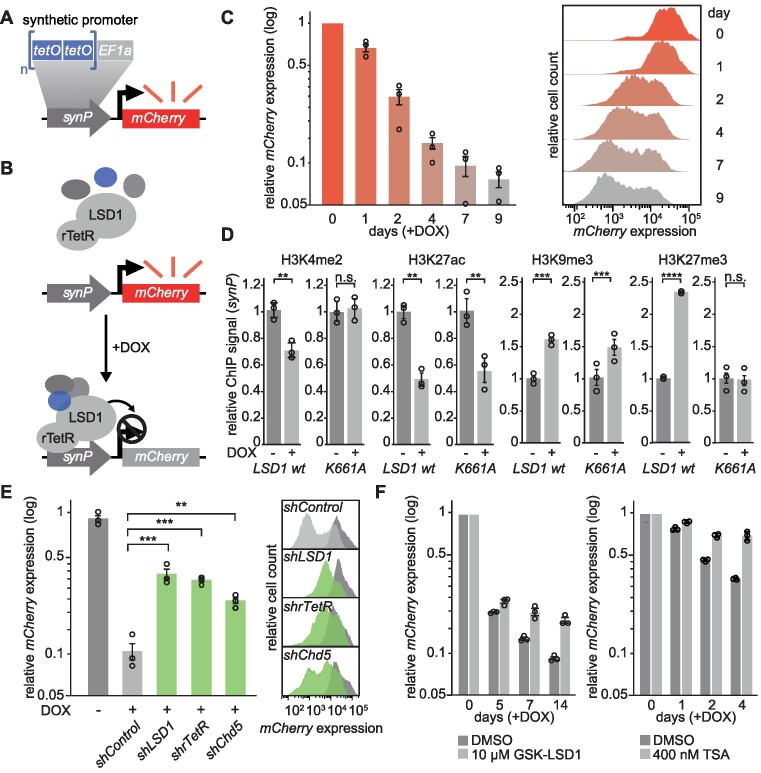
Generation of a fluorescent reporter system to investigate LSD1 and associated cofactors in living cells. (**A**) Illustration depicting the core components of the fluorescent reporter system, which is transduced into cell lines of interest. Stable expression of *mCherry* is driven by a synthetic promoter (*synP*), which consists of 6 Tet repressor binding sites (*tetO*) upstream of an *EF1a* promoter. (**B**) Cell lines expressing the *synP-mCherry* reporter are further transduced with a vector expressing a fusion protein of human LSD1 and the reverse tetracycline repressor protein (rTetR) under a constitutive promoter. Upon DOX treatment, the rTetR-LSD1 fusion protein is recruited to *synP* together with endogenous complex partners, leading to the suppression of *mCherry* expression. (**C**) Flow-cytometric analysis of the *mCherry* expression in NIH/3T3 cells co-expressing the *synP-mCherry* reporter and the rTetR-LSD1 fusion protein after treatment with DOX for the indicated number of days. Left: Bar graphs showing the median *mCherry* signal relative to day 0. Circles represent individual replicates (n = 3, mean±*s.e.m*.). Right: Histograms depicting the distribution of *mCherry* signals of one representative replicate over time (y-axis normalized to highest cell count). (**D**) Bar graphs depicting changes of the indicated histone marks at the *synP* element, analysed by ChIP-qPCR. IPs were performed with mononucleosomes isolated from reporter cell lines at day 4 (H3K4me2, H3K27ac, H3K27me3) or day 14 (H3K9me3) of either LSD1 wt or K661A recruitment. Bar graphs are relative to -DOX. Circles represent independent replicates (n = 3, mean ± s.e.m.; **P ≤ 0.01, ***P ≤ 0.001, ****P ≤ 0.0001, n.s. = non-significant; Student's *t*-test). (**E**) Flow-cytometric analysis of the *mCherry* signal in NIH/3T3 cells expressing the *synP-mCherry* reporter, rTetR-LSD1 and the indicated shRNAs at day 7 of DOX treatment. Dark grey: -DOX, light grey: neutral control shRNA, green: positive control shRNAs +DOX. Left: Bar graphs show the median *mCherry* signal relative to day 0. -DOX is shown for the control shRNA (*shControl)*. Circles represent independent replicates (*n* = 3, mean±*s.e.m*.; **P ≤ 0.01, ***P ≤ 0.001; Student's *t*-test). Right: Histograms showing the *mCherry* expression profiles at day 7 ±DOX of one representative replicate. (**F**) Bar graphs depicting the median *mCherry* expression of +DOX NIH/3T3 reporter cells relative to -DOX in the presence of GSK-LSD1, TSA or DMSO. Treatment of cells was started in parallel with the addition of DOX and maintained for the indicated number of days. Circles represent independent replicates (*n* = 3, mean ± s.e.m.).

The transcriptional output of LSD1 is highly dependent on its associated complex partners. In order to measure the activity of LSD1 in a cellular context and in association with its coregulators in a time-resolved manner, we established a novel reporter system, which can be transduced into cell lines of interest (Figure 1 and Supplementary Figure S1). In this system, the expression of a fluorescent reporter protein (*mCherry*) is driven by a synthetic promoter (*synP*) consisting of six tetracycline repressor (TetR) binding elements (*tetO*) introduced upstream of a strong *EF1a* promoter (Figure 1A).

Following transduction and antibiotic selection, the designed reporter construct exhibited a strong *mCherry* fluorescence signal in different cell lines (Supplementary Figure S1A). Next, we generated a fusion construct of full length human LSD1 with the reverse tetracycline repressor protein (rTetR), which was transduced into NIH/3T3 cells expressing the *synP-mCherry* reporter (Figure 1B). This allowed to induce spatial proximity of rTetR-LSD1 to the *synP* element by the addition of Doxycycline (DOX). Afterwards, LSD1 mediated effects on the *synP-mCherry* reporter gene expression over time can be detected by flow cytometry or fluorescence microscopy (Figure 1B, C and Supplementary Figure S1C, D). ChIP analysis revealed dynamic changes of the chromatin environment at the *synP* promoter element after recruitment of the rTetR-LSD1 fusion protein. We observed a mild reduction in histone H3 lysine 4 dimethylation (H3K4me2) (Figure 1D), which is in agreement with recent literature, stating that the activity of LSD1 is highly dependent on coregulatory effector proteins (22) and probably heavily counterbalanced by H3K4 specific methyltransferases (KMTs) (24,57). Furthermore, this can be explained by the fact that H3K4me2 is an intermediate mark and we observed co-recruitment of Kdm5b (see below), which is an H3K4me3 demethylase that continuously generates H3K4me2 at the locus. Histone H3 lysine 27 acetylation (H3K27ac), another modification characteristic for active chromatin, showed a more pronounced reduction, whereas histone H3 lysine 9 trimethylation (H3K9me3) and H3K27me3, both associated with inactive genomic regions, were increased (Figure 1D). In contrast, the recruitment of rTetR alone to the *synP-mCherry* reporter induced no change in reporter gene expression (Supplementary Figure S1C). Similarly, the recruitment of a catalytically inactive mutant of LSD1 (K661A) (58) to the *synP* promoter did not lead to a strong reduction of fluorescent reporter gene expression when compared to the LSD1 wt (Supplementary Figure S1C). We also investigated the consequences of K661A recruitment on selected histone modifications at the *synP* promoter element. As expected, recruitment of K661A did not lead to a reduction of H3K4me2, whereas changes in H3K27ac or H3K9me3 were comparable to those induced by recruitment of the LSD1 wt (Figure 1D). Interestingly, we did not observe an increase in H3K27me3 when recruiting LSD1 K661A (Figure 1D). These results demonstrated that the observed change in reporter fluorescence is a direct effect of LSD1 recruitment, actively changing the chromatin environment at the promoter and indicate that known coregulators of LSD1, like HDACs and lysine methyltransferases (KMTs) such as G9a/GLP and PRC2, are co-recruited and active at the *synP* element. Furthermore, we observed a mechanistic connection between the enzymatic activity of LSD1 and its ability to induce a strong reduction in gene expression and an increase in H3K27me3, the latter probably related to the inability of PRC2 to methylate histone H3 methylated at K4 (59,60).

To test if the reporter system was sensitive to perturbations and could thus be applied to study the influence of LSD1 associated coregulators, we suppressed the expression of the rTetR-LSD1 fusion construct using shRNAs (Supplementary Data Table S4) targeting the rTetR or LSD1 (Supplementary Figure S1B) parts of the rTetR-LSD1 fusion protein and of the known LSD1 complex partner *Chd5* (61). Silencing of any functional part or *Chd5* resulted in a substantially impaired ability of LSD1 to induce effective silencing of the *synP-mCherry* reporter (Figure 1E). Furthermore, treatment with the LSD1 inhibitor GSK-LSD1 or the pan-HDAC inhibitor Trichostatin A (TSA) demonstrated that the activity of rTetR-LSD1 at the *synP* element is dependent on the catalytic activity of LSD1 and HDACs (Figure 1F and Supplementary Figure S1E). Consequently, silencing of *mCherry* expression by recruitment of rTetR-LSD1 to *synP* is conditioned by the presence and activity of additional endogenous coregulators.

### A multiplexed shRNAmir screen identifies essential and novel LSD1 coregulators

**Figure 2.**
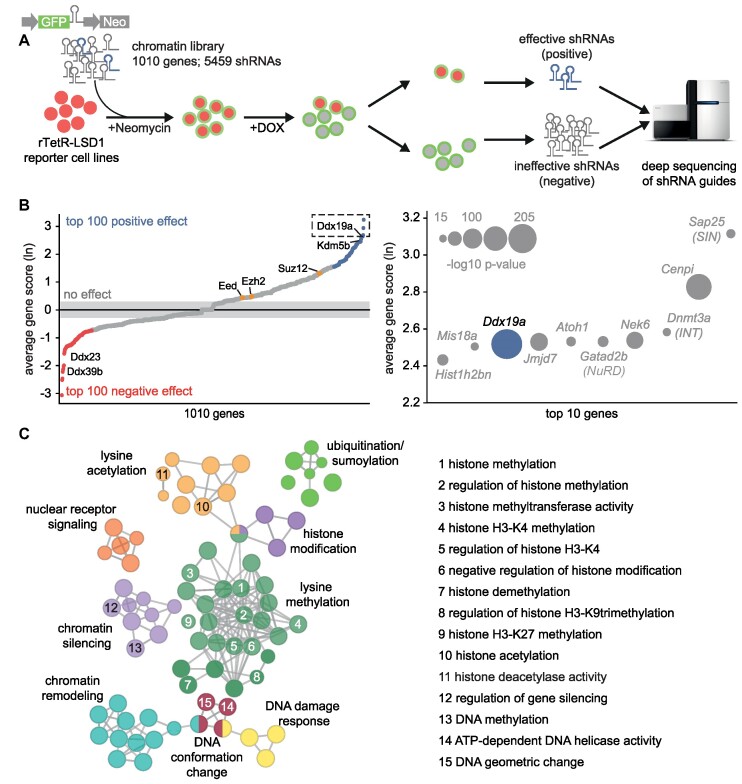
A chromatin-focused shRNA screen identifies novel and known coregulators of LSD1 activity. (**A**) Workflow describing the ChECS screening strategy. A library composed of 5459 shRNAs targeting 1010 chromatin-related genes (*GFP+*) was virally transduced into rTetR-LSD1 reporter cell lines (*mCherry+*). After antibiotic selection, cells were treated with DOX for 14 days and FACS sorted for high (*mCherry+)* or low *(mCherry-) mCherry* expression. Genomic DNA was isolated from both populations and the shRNA guide sequences were amplified for Illumina sequencing. (**B**) Scatter plots ranking all genes according to their effect on LSD1 activity (gene score). Left: Gene scores of all genes present in the shRNA library. The gene score represents the Ln of the average enrichment score (read ratio *mCherry+*/*mCherry-*) of all shRNAs per gene across five replicates. Genes imposing a positive effect on LSD1 induced silencing are coloured in blue, genes having a negative effect are highlighted in red. The position of Ddx19a, Kdm5b and the PRC2 core components Suz12, Ezh2 and Eed is indicated. Right: Top ten genes identified in the screening procedure to positively influence LSD1 activity. Significance is represented by spot size (–log_10_*P*-value). (**C**) *ClueGo* network clustering the top 100 positive and negative regulators of LSD1 for their biological function (GO-annotated biological process). Biological processes of selected clusters are highlighted on the right. The statistical test used for the enrichment was based on a two-sided hypergeometric test with a Bonferroni correction and kappa score of 0.4. Only pathways with p≤0.01 are shown.

We applied our novel reporter system for LSD1 activity to systematically probe a comprehensive selection of chromatin coregulators for their requirement to enable LSD1-mediated silencing. To this end, we screened a focused shRNA library targeting 1010 chromatin-associated murine genes (4–6 shRNAs per gene) in a multiplexed format in NIH/3T3 cells expressing the *synP-mCherry* reporter and the rTetR-LSD1 fusion protein (Figure 2A and Supplementary Table S1). After an initial antibiotic selection for successful integration of the constitutively expressed shRNA constructs, we induced recruitment of the rTetR-LSD1 fusion protein to the *synP* element by the addition of DOX. During subsequent cultivation for 14 days under constant treatment with DOX, cells expressing effective shRNAs targeting regulators of LSD1, which are critically required for LSD1-mediated gene silencing accumulated in a cell population that showed persistent expression of *mCherry* (positive population). Using FACS, these cells were separated from the major population, which exhibited the usual reduction in fluorescent reporter signal (negative population) and expressed ineffective shRNAs (Figure 2A and Supplementary Figure S2A). The representation of each shRNA in the input library and in the sorted positive and negative cell populations was quantified using deep-sequencing of the shRNA guide strands amplified from genomic DNA using established protocols (Supplementary Figure S2B and Table S1) (62). To rank all genes represented in the shRNA library for their effect on LSD1 activity, gene scores reflecting the enrichment of multiple shRNAs per gene in the positive cell population compared to the negative population were calculated (Figure 2B, and Supplementary Table S2). As expected, the screen managed to identify coregulators of LSD1 that were already described as complex partners in the literature (Figure 2B) and suppression of several genes known to be associated with LSD1, e.g. *Dnmt3a* (63), *Gatad2b* (NuRD) (64) and *Sap25* (SIN) (65,66), were confirmed to be especially important for LSD1-mediated gene silencing. Interestingly, the H3K4me3 demethylase Kdm5b was ranked at position 20 in the screen, suggesting that LSD1 requires the demethylase activity of KDM5B (67) to generate K4me2, which then can be demethylated further by LSD1 leading to stable silencing of the *synP-mCherry* reporter in NIH/3T3 cells. In addition to already known factors, the screen also identified novel coregulators not associated with LSD1 biology so far (Figure 2B and Supplementary Figure S2B).

We selected the top 100 positive and negative hits identified in the screen and performed an enrichment analysis using ClueGO (68) to visualize functionally related coregulators influencing LSD1 activity as a clustered network of the associated Gene Ontology (GO) pathways (Figure 2C). These data show that LSD1 mainly cooperates with proteins linked to biological pathways associated with lysine methylation including pathways regulating H3K4, K9 or K27 methylation, which constitutes the largest cluster in the network (Figure 2C). Other clusters comprise pathways associated with chromatin silencing, lysine acetylation and chromatin remodeling. Furthermore, the network is enriched for pathways related to DNA damage response and nuclear receptor signaling, functions which were already described to be regulated by LSD1 (7,17,32).

Interestingly, one cluster in the network is connected to conformational changes of DNA (Figure 2C) and indeed the screen identified three ATP-dependent RNA helicases among the top 10 positive and negative coregulators of LSD1 (Figure 2B). DDX39B, DDX23 and DDX19A belong to the so-called DEAD-box family of RNA-dependent ATPases that have RNA unwinding activity and are involved in pre-mRNA splicing, mRNA export from the nucleus to the cytoplasm or translation (69–73). Loss of DDX39B and DDX23 promoted silencing of the *synP* element, likely due to LSD1 unrelated effects caused by deregulation of the transport, splicing or translation of components of the reporter system, functions reported for both helicases previously (72,74). Among the three DEAD-box helicases identified as coregulators, DDX19A was the only helicase identified as a positive regulator of LSD1 mediated silencing and it scored with the highest significance in the screen (Figure 2B). The homolog of DDX19A, DDX19B has recently been shown to be involved in the removal of RNA:DNA hybrid structures (so-called R-loops) and the activity of DDX19B was shown to be dependent on the DNA damage response induced by the ATR‐Chk1 pathway (54).

R-loops are highly dynamic structures that occur at different regions in the eukaryotic genome and exhibit critical regulatory functions during replication, transcription, and recombination (75–78). R-loops preferentially form at GC-rich regions, where the newly synthesized G-rich RNA hybridizes to the C-rich DNA template (79). They have been described to be associated with both up- and downregulation of transcription (80) and occur at unmethylated human CpG island promoters (81). Interestingly, R-loops have been shown to colocalize with H3K4 methylation on a genome-wide scale (82).

Aiming to characterize the role of DDX19A in LSD1 induced silencing of gene expression, we further investigated the effects of *Ddx19a* suppression on R-loop regulation and gene expression on a global and local level. Two top-scoring shRNAs from our screen (*shDdx19a*.1/2) showed only mild effects on cell viability, suppressed DDX19A expression (Supplementary Figure S2C, D) and were validated to interfere with LSD1 silencing activity at the *synP* promoter (Supplementary Figure S2E). We next sought to determine the mechanism by which DDX19A influences LSD1-induced silencing and analysed rTetR-LSD1 protein levels after suppression of *Ddx19a* to rule out that the effect of *Ddx19a* is merely a consequence of reduced expression or defects in mRNA processing of the rTetR-LSD1 fusion protein. However, we did not observe any reduction in expression of the rTetR-LSD1 fusion protein and no alteration of the expression of the *synP-mCherry* reporter without the addition of DOX, which could lead to a false-positive result and be responsible for the observed remaining fluorescence signal (Supplementary Figure S2F, G). To confirm that the negative effect of *Ddx19a* suppression of LSD1-mediated silencing is specific to LSD1 function, we investigated the effects of *Ddx19a* suppression on the repressive activity of KRAB, which we recruited to the *synP* element using a rTetR-KRAB fusion protein. Suppression of Ddx19a expression did not influence the activity of KRAB, suggesting that DDX19A is not a general requirement for gene silencing and the observed function is specific to LSD1 (Supplementary Figure S2H).

DDX19A has not been described as an interactor of LSD1 (83,84) and to test if LSD1 recruits DDX19A by a direct interaction, we performed co-immunoprecipitation (co-IP) experiments from NIH/3T3 cell lysate. Although other known complex partners of LSD1 like HDAC1 co-precipitated with LSD1, we could not detect a direct interaction of LSD1 and DDX19A (Supplementary Figure S2I), which could be due to low abundance of DDX19A or a weak interaction with LSD1. Thus, we aimed to further characterize the effects of *Ddx19a* suppression on LSD1 activity in more detail.

### DDX19A is involved in R-loop homeostasis

**Figure 3.**
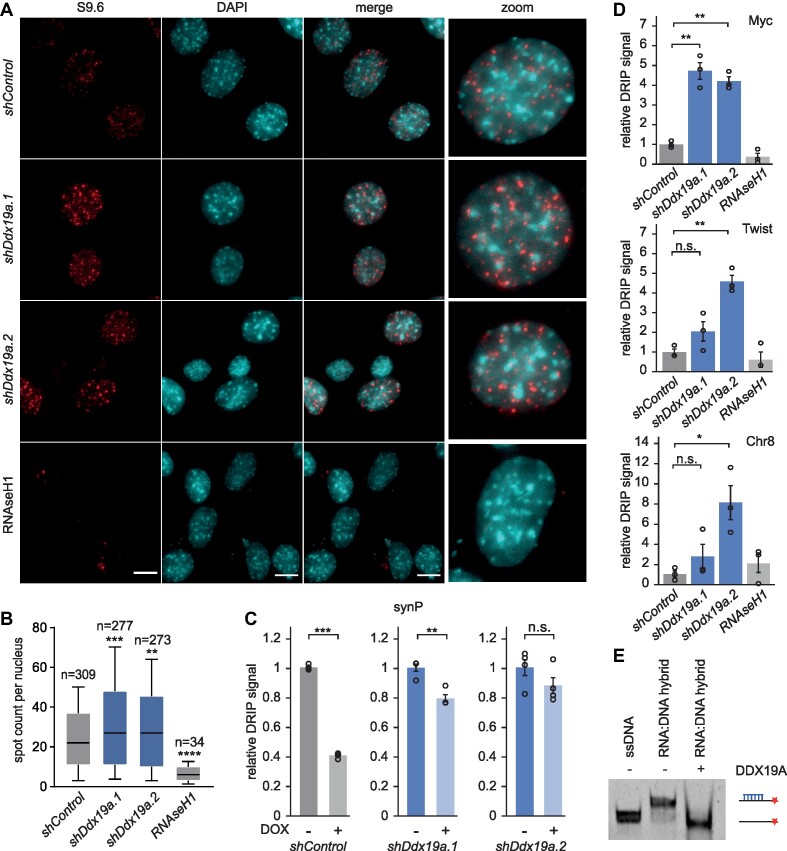
DDX19A is involved in R-loop homeostasis. (**A**) Representative immunofluorescence microscopy images of NIH/3T3 cells expressing the indicated shRNAs for 10 days and stained for R-loops with the S9.6 antibody. Transfection with RNAseH1 24 h before fixation serves as a control for the S9.6 antibody specificity. Images are maximum intensity projections of a Z-stack covering the whole nucleus. Scale bars are 10 μm. (**B**) Box and whisker plot showing the quantification of the R-loop spot count per nucleus in images from (**A**). Images were analysed using CellProfiler™ software. Box-and-Whisker plots indicate the median and the 10–90 percentile from three independent experiments (**P ≤ 0.01, ***P ≤ 0.001, ****P ≤ 0.0001, Student's *t*-test). (**C**) DRIP-qPCR analysis of RNA:DNA hybrid structures at the *synP* element upon recruitment of rTetR-LSD1 and under suppression of *Ddx19a*. Total nucleic acids were extracted from NIH/3T3 cells expressing the *synP-mCherry* reporter, the rTetR-LSD1 fusion protein and the indicated shRNAs at day 14 with and without DOX treatment and used as input for the IP with the S9.6 antibody. qPCR signals are shown relative to -DOX. Circles represent independent replicates (*n* = 4, mean ± s.e.m.; **P ≤ 0.01, ***P ≤ 0.001, n.s. = non-significant, Student's *t*-test). Pre-treatment of the input samples with RNAseH1 before DRIP was used as a negative control. (**D**) DRIP-qPCR analysis of RNA:DNA hybrid structures at selected endogenous loci under suppression of *Ddx19a* expression. Total nucleic acids were extracted from NIH/3T3 cells expressing the *synP-mCherry* reporter, the rTetR-LSD1 fusion protein and the indicated shRNAs for 10 days and used as input for the IP with the S9.6 antibody. qPCR signals are shown relative to *shControl*. Circles represent independent replicates (*n* = 3, mean ± s.e.m.; *P ≤ 0.05, **P ≤ 0.01, n.s. = non-significant, Student's *t*-test). (**E**) Representative result of an *in vitro* RNA:DNA hybrid unwinding assay using recombinant DDX19A. On the right of the image, the Cy5-labeled single strand DNA (ssDNA) and the RNA:DNA hybrid substrate are shown schematically. The RNA is colored blue, the DNA black and the Cy5 label is indicated with a red star.

Since the homologue of DDX19A, DDX19B, actively participates in the removal of R-loops (54), we investigated the dynamics of R-loop formation in NIH/3T3 cells after suppression of *Ddx19a* using immunofluorescence microscopy. We employed the S9.6 antibody, which specifically recognizes R-loops (85). To test for the specificity of the signal detected with the S9.6 antibody, we used Ribonuclease H1 (RNAseH1), known to specifically degrade the RNA of RNA:DNA hybrid structures. As shown in Figure 3A, transient expression of RNAseH1 prior to the immunostaining with the S9.6 antibody led to a strong reduction of the R-loop signal (Figure 3A). Upon suppression of *Ddx19a* we noticed a significant increase in R-loop spot counts per nucleus compared to a neutral control shRNA (Figure 3A, B and Supplementary Figure S3A) as well as an enhancement of R-loop spot intensity (Figure 3A and Supplementary Figure S3A, B). Subsequently, we studied the alterations of R-loops at the *synP* element before and after DOX induced LSD1 recruitment. As expected, R-loops at the *synP* promoter element were reduced following DOX induced recruitment of LSD1 and this effect was strongly attenuated under suppression of *Ddx19a* (Figure 3C). Furthermore, we investigated R-loop dynamics at representative endogenous genomic loci (Supplementary Figure S3E) upon suppression of *Ddx19a* expression. Two regions are associated with the developmental master regulators Myc (86,87) and Twist1 (88,89), which are highly expressed and associated with extensive H3K4 methylation and R-loop signal. In addition, we investigated R-loop dynamics at an intergenic region on chromosome 8 (Chr8) characterized by very low R-loop and high H3K27me3 signal (Supplementary Figure S3E). Using DNA-RNA immunoprecipitation (DRIP) followed by qPCR, we observed an increase in R-loops upon *Ddx19a* suppression in all cases (Figure 3D) showing that also master regulators like Myc and Twist1 as well as regions decorated with high levels of H3K27me3 respond in a similar way to suppression of *Ddx19a* expression as the *synP* element of our artificial reporter construct. As a control, we included an RNAseH1 incubation step, which reduced the DRIP signal confirming the specificity of the antibody (Figure 3D and Supplementary Figure S3C, D). This is in line with our observations in the immunofluorescence experiments and implies that the effect of suppression of *Ddx19a* expression on R-loops is not restricted to our artificial reporter construct but also occurs at endogenous regions.

In addition, we purified recombinant DDX19A and confirmed its ability to resolve RNA:DNA hybrids *in vitro* (Figure 3E and Supplementary Figure S3F). Our data indicate that suppression of Ddx19a expression leads to the accumulation of R-loops and interferes with the silencing activity of LSD1 at the *synP-mCherry* reporter. This observation is in agreement with the model that R-loops forming over the *synP* element stabilize its transcriptional activity, which is supported by recent publications showing that R-loops impose various effects on chromatin regulators to promote transcription (81,82,90–93) including the inhibition of LSD1 (38) and PRC2 (39,40).

### LSD1 and R-loops colocalize and occupy regions associated with highly transcribed genes

**Figure 4.**
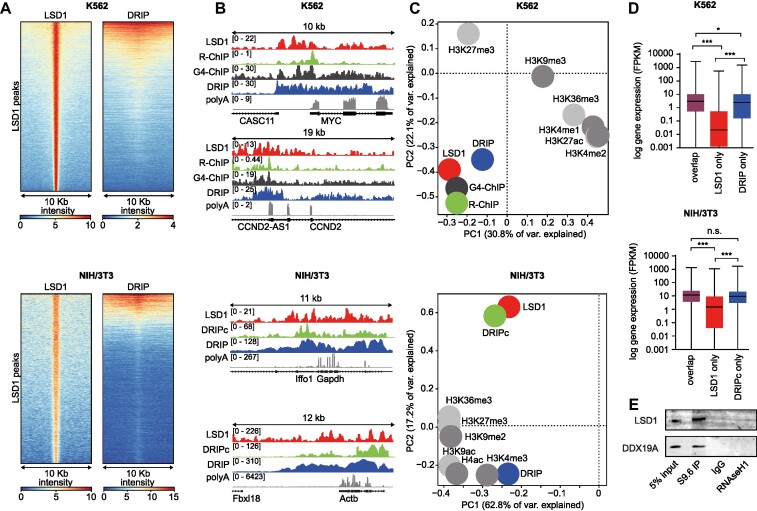
Regions of LSD1 occupancy and R-loop related features correlate genome wide in K562 and NIH/3T3 cells. (**A**) Heatmaps of LSD1-ChIP and DRIP signals in K562 (top) and NIH/3T3 cells (bottom). Signals are plotted on the heatmap within a 10 kb window around the peak centre. (**B**) Representative regions showing the occupancy of R-loop related features and LSD1 in K562 and NIH/3T3 cells. (**C**) Principle component analysis of deep sequencing data for K562 (top) and NIH/3T3 (bottom) cells. ChIP-seq, DRIP-seq, DRIPc-seq, R-ChIP-seq and G4-ChIP-seq datasets were obtained from the Gene Expression Omnibus (GEO) repository and the ENCODE project. R-loop related features are highlighted in blue, green and black. R-loop features analysed in panel B and C: RNA:DNA hybrid IP (DRIP); RNA:DNA hybrid IP followed by cDNA conversion (DRIPc); RNA:DNA hybrid IP using a catalytically inactive RNAseH1 (R-ChIP); Genome-wide mapping of endogenous G-quadruplex DNA structures (G4-ChIP). (**D**) Expression levels (mRNA, FPKM) of LSD1- and DRIP/DRIPc-associated genes in K562 and NIH/3T3 (*P ≤ 0.05, **P ≤ 0.001, n.s. = non-significant, ordinary one-way ANOVA with multiple comparisons test). (**E**) Representative Western blot showing co-precipitation of LSD1 and DDX19A with R-loops after IP using the S9.6 antibody. NIH/3T3 whole cell lysate was used as input. Pulldown with mouse IgG and treatment with RNAseH1 before IP with the S9.6 antibody was used as control.

We wanted to explore if the interaction of LSD1 and DDX19A could be mediated through R-loops. In order to investigate if LSD1 localizes to genomic regions decorated with R-loops, we analysed the genome-wide distribution of LSD1 in two different cell lines (K562 and NIH/3T3) and compared it to features associated with R-loops and histone modifications signaling either active or repressed gene expression (Figure 4 and Supplementary Figure S4). To this end, we performed an LSD1 ChIP-seq in NIH/3T3 cells, employed publicly available LSD1 ChIP-seq data from K562 cells (94) and compared those to available DRIP-seq datasets that investigated the genome-wide distribution of R-loops using the S9.6 antibody (82). This analysis revealed a considerable colocalization of LSD1 with R-loops in both cell lines (Figure 4A). We further investigated the colocalization of LSD1 with additional R-loop features and used publicly available data of an IP with a catalytically dead RNAseH1 mutant that specifically binds to R-loops (R-ChIP-seq) (95) or a G-quadruplex (G4) specific antibody (G4-ChIP-seq) (96) and compared these with ChIP-seq datasets of different histone modifications from the Gene Expression Omnibus (GEO) repository (97) and the ENCODE project (98) for both cell lines (see Materials and Methods section for the corresponding GO numbers). Surprisingly, LSD1 binding showed a much stronger correlation with R-loop related features than with histone modifications previously reported to associate with LSD1, like methylated H3K4 (22) in both cell lines (Figure 4B, C and Supplementary Figure S4A, B).

Expression of the genes showing LSD1 binding and/or R-loop features was retrieved from previously published RNA-seq analysis for K562 (62) and publicly available RNA-seq data for NIH/3T3 cells from ENCODE. A detailed analysis of the expression of genes positioned in the vicinity of either LSD1 binding or R-loop structures showed that genes which were solely bound by LSD1 are expressed at a very low level. In contrast, loci which are only decorated with features of R-loops were associated with genes exhibiting high expression levels in both cell lines (Figure 4D and Supplementary Figure S4C–E). However, genomic regions covered with LSD1 and R-loops together were affiliated with genes expressed at an even higher level than genes associated with R-loops only (Figure 4D and Supplementary Figure S4C–E). Next, we performed a gene ontology (GO) enrichment analysis of these genes for the K562 cell line to analyse these genes according to their annotated biological process. We identified ‘negative regulation of erythrocyte differentiation’ among the top 3 enriched pathways ranked for the respective *P*-value (Supplementary Figure S4F), which is in line with the fact that the chronic myelogenous leukaemia (CML) cell line K562 bears resemblance with undifferentiated erythrocytes (99).

To support these global observations, we performed an immunoprecipitation of R-loops from NIH/3T3 cell lysate using the S9.6 antibody, followed by immunodetection of LSD1 and DDX19A, which indicated a strong physical association of both proteins with R-loops. Co-precipitation of LSD1 or DDX19A was attenuated if the sample was treated with RNAseH1 before the IP (Figure 4E). These findings imply that LSD1 and R-loops co-occupy highly transcribed genes, which are important to control cell type-specific developmental gene expression profiles and define cellular identity. This is in agreement with previous reports showing that, despite its mostly repressive function, LSD1 occupies enhancers and core promoters of a substantial fraction of actively transcribed genes (28) and suggests that the activity of LSD1 at these regions is repressed.

### DDX19A plays a role in LSD1 induced silencing

**Figure 5.**
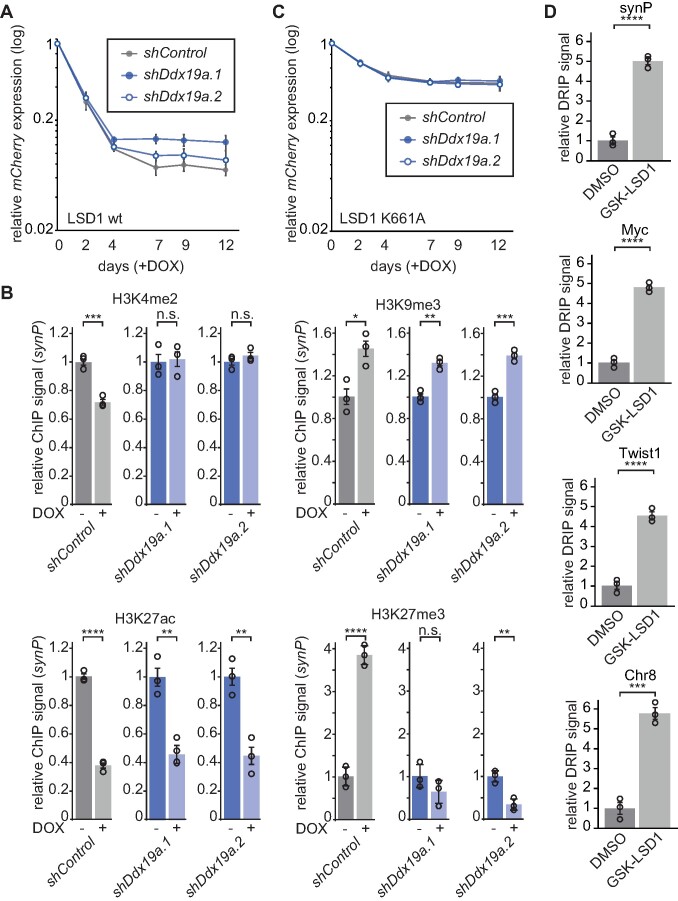
LSD1 and DDX19A act in concert to regulate transcription. (**A**) Time course of the *mCherry* expression in NIH/3T3 expressing the *synP-mCherry* reporter, the rTetR-LSD1 fusion protein and the indicated shRNAs under DOX treatment. Circles indicate the median *mCherry* expression measured by flow cytometry relative to the initial measurement (*n* = 3; mean ± s.e.m.). (**B**) ChIP-qPCR analysis of the indicated histone modifications at the *synP* element in cells expressing the LSD1 reporter system and the indicated shRNAs. IPs were performed with mononucleosomes isolated from NIH/3T3 reporter cell lines after 4 days (H3K4me2, H3K27ac) or 14 days (H3K9me3) of LSD1 recruitment. Bars are relative to -DOX (*n* = 3; mean ± s.e.m.; *P ≤ 0.05, **P ≤ 0.01, ***P ≤ 0.001, ****P ≤ 0.0001, n.s. = non-significant; Student's *t*-test). (**C**) Time course of the *mCherry* expression in NIH/3T3 cells expressing the *synP-mCherry* reporter, the rTetR-K661A fusion protein and the indicated shRNAs under treatment with DOX. Circles indicate the median *mCherry* expression measured by flow cytometry relative to day 0 (*n* = 3; mean ± s.e.m.). (**D**) DRIP-qPCR analysis of RNA:DNA hybrid structures at selected endogenous loci under treatment with GSK-LSD1. Total nucleic acids were extracted from NIH/3T3 cells transduced with the *synP-mCherry* reporter after 3 days of treatment with 50 μM GSK-LSD1 or DMSO and used as input for the IP with the S9.6 antibody. qPCR signals are shown relative to DMSO. Circles represent independent replicates (*n* = 3, mean ± s.e.m.; ***P ≤ 0.001, ****P ≤ 0.0001, Student's *t*-test).

To gain additional insights into the local consequences of R-loop accumulation, in particular the effects on downstream effector proteins of LSD1, we analysed histone modifications at the *synP* promoter element after suppression of *Ddx19a*, which led to an incomplete silencing of the *synP-mCherry* reporter after recruitment of LSD1 (Figures 2B, Supplementary Figure S2E and 5A). Despite a significantly diminished reduction of H3K4me2 (Figure 5B), which was comparable to the level induced by the catalytically inactive LSD1 mutant K661A (Figure 1D), we did not observe any alteration in H3K9me3 and H3K27ac histone modifications after suppression of *Ddx19a* expression compared to *shControl* (Figure 5B). In contrast, we noticed a distinct reduction in the increase of H3K27me3 signal at the *synP* element upon LSD1 recruitment and suppression of *Ddx19a* (Figure 5B). These results suggest, that the increase in R-loops at the *synP* promoter (Figure 3C) influences the removal of H3K4me2 and/or deposition of H3K27me3, thereby interfering with efficient reduction of gene expression. This effect can be explained by the inhibition of LSD1 (38) and PRC2 (39,40) via RNA that is anchored at the locus through R-loops. This interpretation is in line with the fact that suppression of *Ddx19a* expression did not affect the residual silencing activity of K661A (Figure 5C). Interestingly, we observed a substantial increase in R-loops at the *synP* element and representative endogenous regions upon treatment with GSK-LSD1, a selective LSD1 inhibitor (Figure 5D and Supplementary Figure S5), demonstrating that inhibition of LSD1 activity results in a gain of R-loops not only at our artificial promoter element but also representative endogenous loci, which suggests a mutual influence of LSD1 and R-loops on one another.

### DDX19A binds to defined histone modifications associated with gene repression

**Figure 6.**
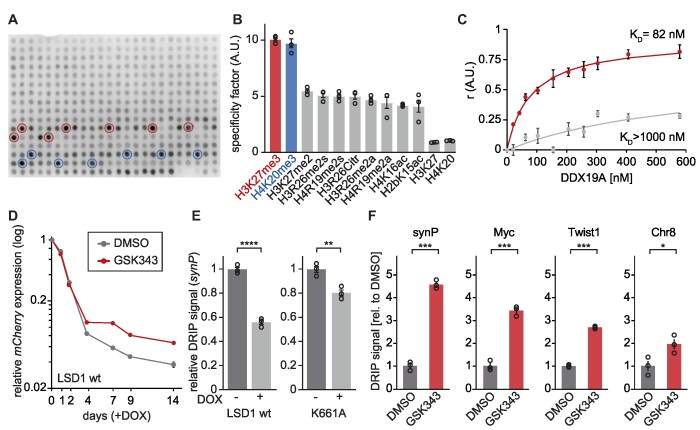
H3K27me3 provides a binding site for DDX19A and regulates the formation of R-loops. (**A**) Representative MODified™ Histone Peptide Array incubated with 50 nM DDX19A. Peptides featuring H3K27me3 are highlighted in red, peptides featuring H4K20me3 in blue. (**B**) Bar graph comparing signals of the top 10 histone modifications bound by DDX19A to binding of DDX19A to the unmodified peptides. DDX19A binding was quantified on independent MODified™ Histone Peptide Arrays using the Array Analyze software (*n* = 3; mean ± s.e.m.). ‘H3K27’ and ‘H4K20’ refers to the specificity factor of the corresponding unmodified peptides. [A.U.] = arbitrary unit. (**C**) Determination of the dissociation constant of DDX19A binding to H3K27me3 (red) or H3K27me0 (gray) by equilibrium peptide binding experiments. Fluorescence anisotropy measurement was performed with a fluorescein (FITC)-labeled peptide incubated with recombinant DDX19A. [A.U.] = arbitrary unit. (**D**) Time course of *mCherry* expression in NIH/3T3 expressing the *synP-mCherry* reporter during recruitment of the rTetR-LSD1 fusion protein via DOX in the presence of 5 μM GSK343 or DMSO. Circles indicate the median *mCherry* expression measured by flow cytometry relative to the initial measurement (*n* = 3; mean ± s.e.m.). (**E**) DRIP-qPCR at the *synP* element after recruitment of LSD1 wt or K661A via DOX addition. Total nucleic acids for the IP were isolated after 14 days of DOX treatment. Bars are relative to -DOX. Circles represent independent replicates (*n* = 3, mean ± s.e.m., **P ≤ 0.01, ****P ≤ 0.0001, Student's *t*-test). (**F**) DRIP-qPCR analysis of RNA:DNA hybrid structures at the indicated regions after the treatment of cells with 5 μM GSK343 or DMSO for 3 days. Total nucleic acids were extracted from NIH/3T3 cells expressing the *synP-mCherry* reporter and used as input for IP with the S9.6 antibody. qPCR signals are shown relative to DMSO. Circles represent independent replicates (*n* = 3, mean ± s.e.m.; *P ≤ 0.05, ***P ≤ 0.001, n.s. = non-significant, Student's *t*-test).

Investigating the downstream consequences of impaired H3K4 demethylation at the *synP* promoter element, we noticed that the K661A inactive mutant was ineffective in inducing a gain of H3K27me3 signal in contrast to the LSD1 wt (Figure 1D). This led to the conclusion that the reduction of H3K4me2 allows PRC2 to bind to the *synP* promoter (59,60). The fact that suppression of *Ddx19a* expression did not affect residual gene silencing by the K661A mutant suggests that the activity of LSD1 is a prerequisite for DDX19A to facilitate a robust reduction of transcription. While, the exact mode how DDX19A is recruited to the *synP* promoter element remained unknown, the previous results suggested that the gain of H3K27me3 seemed to play an important role in this process (Figures 1D and 5B). Searching for the underlying mechanism by which LSD1 activity can induce the recruitment of DDX19A, we screened the binding properties of recombinant full-length DDX19A (Supplementary Figure S3F) to 384 different histone peptides containing 59 post-translational modifications of the N-terminal tails of histones H2A, H2B, H3 and H4. Although DDX19A does not contain any of the known domains specific for lysine methylation binding, we observed a remarkably distinct interaction of DDX19A with H3K27me3 and to a lesser extent H4K20me3 among all other tested histone modifications (Figure 6A, B and Supplementary Figure S6A). We validated this effect with additional modified histone tail peptide arrays containing selected methylated and unmethylated lysine residues on H3 and H4 (Supplementary Figure S6B). Using fluorescence anisotropy (FA), we determined the dissociation constant (*K*_D_) of DDX19A with the H3K27me3 peptide with 82 (±2.9) nM while the corresponding unmethylated peptide showed a much weaker affinity with a *K*_D_ >1000 nM (Figure 6C).

The role of H3K27me3 in the recruitment or stimulation of DDX19A was confirmed by inhibition of the core PRC2 complex component EZH2 using an EZH2-specific inhibitor (GSK343), which induces a global reduction of H3K27me3 signal (Supplementary Figure S6C). Indeed, we observed increased R-loop occupancy at the *synP* element and representative endogenous loci after GSK343 treatment confirming the reduced activity of DDX19A at the *synP* element (Figure 6F, Supplementary Figures S3D and S6D). As a consequence of reduced DDX19A binding to H3K27me3 and elevated R-loop occupancy, GSK343 treatment resulted in a diminished LSD1 silencing capacity at the *synP* element (Figure 6D) comparable to the activity under suppression of DDX19A (Figure 5A). Furthermore, K661A did not reduce R-loops at the *synP* element as efficiently as the LSD1 wt (Figure 6E) after recruitment to the *synP* element, likely due to the lack of H3K4 demethylation preventing deposition of H3K27me3. As expected by the reduced DDX19A activity or recruitment under these conditions, residual silencing of the *synP* element by LSD1-K661A was not affected by the suppression of *Ddx19a* expression (Figure 5C). We conclude that the DEAD-box helicase DDX19A specifically interacts with histone modifications signaling repressive chromatin states, preferably H3K27me3, which is introduced by PRC2 upon reduction of H3K4 methylation by LSD1 (Figure 1D). This binding precedes the ATP-dependent helicase activity of DDX19A regulating the formation of R-loops (Figure 6D, E).

## DISCUSSION

Transcription is regulated by a complex network of coregulators often with opposing functions, which act in concert to fine tune gene expression. LSD1 has been demonstrated to be an important regulator of developmental genes in embryonic stem cells and malignant cells (9,28) and it was shown to interact with a large variety of different coregulator complexes. The mechanisms underlying the precise regulation of the pluripotency program and its response to developmental cues are still relatively unknown. The tightly controlled balance of opposing chromatin effector functions such as KMTs, lysine demethylases (KDMs) or histone acetyltransferases (HATs) and HDACs (100,101) constitutes a feasible mechanism to ensure developmental plasticity (102) and several reports demonstrate that LSD1 and the PRC2 complex are associated with actively transcribed developmental genes (28,103,104). To explore these functional networks in detail we adopted the concept of the CiA assay (44) and investigated the function of epigenetic effector proteins in LSD1 mediated gene silencing. By fusing LSD1 to rTetR, we induced spatial proximity of LSD1 with a synthetic promoter element (*synP*) consisting of six *tetO* sites upstream of an *EF1a* promoter through the addition of DOX. The reporter system is modular, highly flexible and allows to study epigenetic effectors in different cell lines of various cell types in a time-resolved manner independent of a specific genomic locus (Figure 1 and Supplementary Figure S1). We combined the fluorescent reporter system for LSD1 activity with a comprehensive library of shRNAs targeting chromatin associated proteins and performed a chromatin effector coregulator screen (ChECS) to identify functional, essential and novel coregulators. Compared to screening methods that depend on the temporal and chemical stability of physical complexes of two or more proteins, the ChECS approach enables the identification of coregulatory factors on the basis of a functional connection between the coregulator and the target factor, in this study LSD1. This allows to detect novel dependencies in epigenetic networks (Figure 2) in an unbiased fashion without the need for a pre-existing hypothesis. One of the top hits identified in our screen, the DEAD-box helicase DDX19A, had not been connected to LSD1 biology before. Following up on the identification of DDX19A, we showed that it plays an important role in LSD1 induced silencing and attributed this role to its function in R-loop homeostasis (Figure 3 and Supplementary Figure S3). Suppression of *Ddx19a* expression led to a strong accumulation of R-loops in the nuclei of NIH/3T3 cells leading to stabilization of the fluorescent reporter protein expression after recruitment of LSD1 (Figure 2B, Supplementary Figure S2E and Figure 5A).

**Figure 7.**
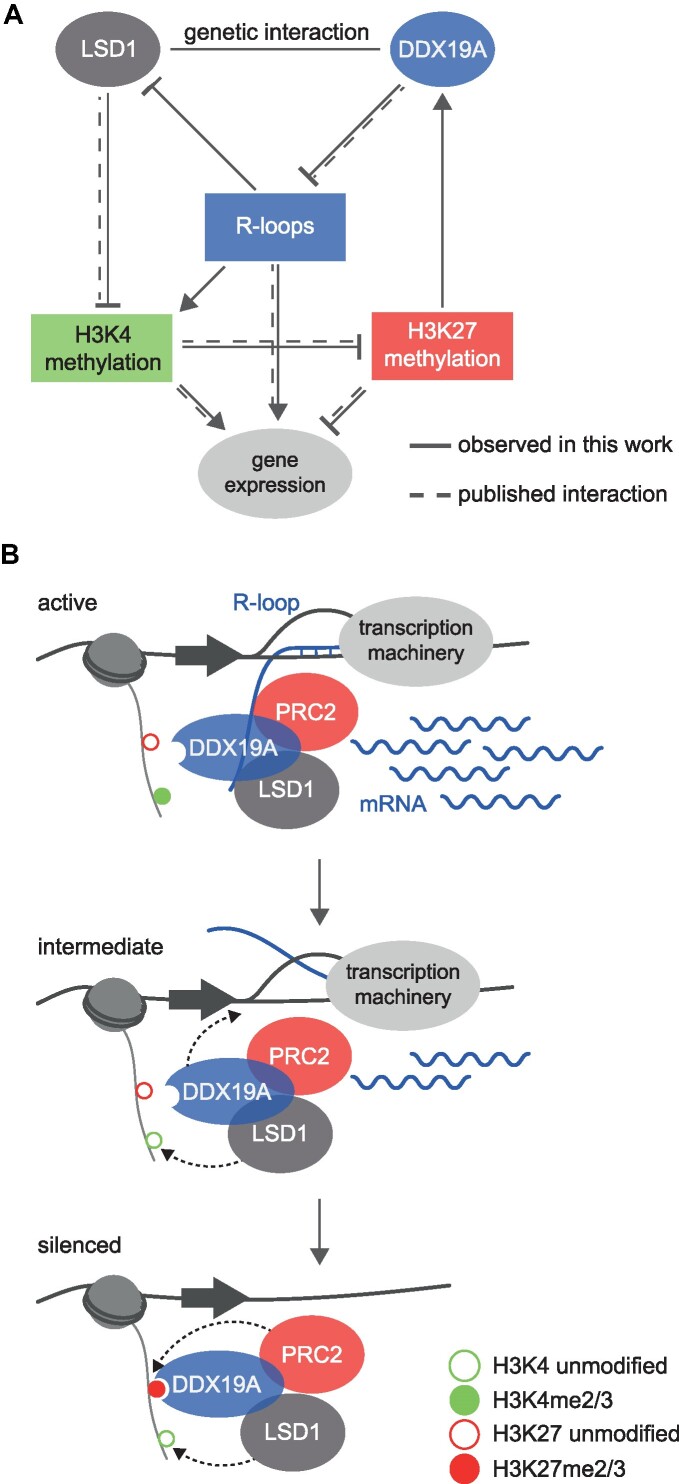
Expression of actively transcribed genes is regulated by a network of histone modifications and R-loops. (**A**) Regulatory network depicting the known and novel connections that interact to modulate gene expression. (**B**) Model of the regulatory cascade for transcriptional repression depending on the removal of transcription-associated R-loops downstream of LSD1 activity. LSD1 and PRC2 are localized at highly transcribed genes. Active transcription promotes the local presence of H3K4me2/3 and the formation of R-loops. These R-loops are balanced by specific helicases (e.g. DDX19A). Upon an external repressive stimulus LSD1 activity is increased leading to a reduction in H3K4 methylation, which enables the PRC2 complex to methylate H3K27. H3K27me3 serves as a binding motif for DDX19A, which then efficiently removes local R-loops and leading to robust silencing of transcription.

Our results expand the knowledge of the highly complex regulatory network surrounding LSD1 (Figure 7A) and identified a novel regulatory circuit embedded in this network, which enables robust downregulation of transcription upon an external stimulus. Our results indicate that LSD1 and PRC2 are prevented from gene silencing through R-loops or the associated nascent RNA, which is in line with recent publications that already described an inhibitory effect on LSD1 and the PRC2 complex through specific RNA structures (38–40). If a cell perceives an external signal, e.g. during development, the expression of developmental genes needs to be completely and robustly repressed in order to prevent uncontrolled proliferation (105,106). During this process R-loops, which are not only a consequence of transcription but also pose a regulatory function (80,107), need to be removed in order to allow robust gene silencing. Removal of R-loops can be triggered by the recruitment of DDX19A, or the activation of the already bound enzyme. Currently it is unclear what guides this initial recruitment, but the fact that DDX19 is a helicase able to bind to RNA (108) suggests that DDX19A can be recruited to sites of active transcription by the nascent RNA. The removal of R-loops by DDX19A activates LSD1 leading to the demethylation of H3K4, presumably in concert with KDM5b, another top hit of our screen. H3K4me2/3 demethylation is a prerequisite for the methylation of H3K27 by PRC2 in our system, which is in line with published literature (59,60). Subsequently, the introduced H3K27me3 signal provides a binding motif for DDX19A, which enhances DDX19A activity by reinforcing the binding to target loci or amplifying the enzymatic activity of DDX19A to effectively remove remaining R-loops (Figure 7B). This activity finally increases the activation of LSD1 and PRC2 establishing a self-enforcing feedback cycle. Therefore, the entire sequence of events is required to induce a strong and stable shut-down of gene expression and in case one of the components is lost or inactive, the silencing stimulus will not lead to a complete reduction of expression of the respective gene (Figure 7). In the ChECS system, reporter gene silencing was triggered by the recruitment of LSD1. During development or differentiation, silencing of endogenous loci could be a result of changes in the catalytic activity of LSD1, PRC2 or it could be triggered by changes in expression of associated genes.

This novel mechanism is supported by the following key observations described previously in detail: (i) The biochemical activity of DDX19A in resolving R-loops was demonstrated *in vitro* and silencing of *Ddx19a* expression was shown to lead to an increase in R-loop occupancy at the reporter gene and endogenous loci. (ii) We observed a correlation of LSD1 occupancy with the distribution of R-loop associated features genome-wide via the analysis of ChIP-seq data in two different cell lines (Figure 4 and Supplementary Figure S4), illustrating that LSD1 and R-loops co-occupy regions in the vicinity of highly expressed genes (Figure 4D and Supplementary Figure S4E) that are associated with essential developmental transcription programs (Supplementary Figure S4F). Furthermore, a direct physical interaction of LSD1 and DDX19A with R-loops was confirmed by co-IP using a RNA:DNA hybrid-specific antibody (Figure 4E). (iii) R-loop enrichment by *Ddx19a* suppression affected the robust downregulation of transcription only if LSD1 was catalytically active suggesting that R-loops inhibit LSD1 enzymatic activity (Figure 5A, C). Suppression of *Ddx19a* expression did not only lead to an increase in R-loops but also to an impaired demethylation of H3K4me2 and reduced increase in H3K27me3. (iv) Reduction of LSD1 activity by recruitment of an inactive mutant or application of a small molecule inhibitor, not only led to reduced decline of H3K4me2, but also reduced introduction of H3K27me3 and increased R-loop occupancy. The observation that H3K27 methylation by PRC2 depends on H3K4me2 demethylation is in line with published literature (59,60). (v) Application of a PRC2 inhibitor not only prevented introduction of H3K27me3, but also led to an increase in R-loops and it impedes reporter gene silencing. (vi) H3K9me3 deposition and H3K27 deacetylation following LSD1 recruitment were not affected by suppression of *Ddx19a* suggesting that known complex partners of LSD1 like G9a and HDACs were still recruited (Figure 5B). (vii) We observed distinct binding of DDX19A to H3K27me3 and H4K20me3 histone peptides (Figure 6A–C and Supplementary Figure S6A, B) providing biochemical evidence for the interaction of DDX19A with H3K27me3.

Taken together, we describe a novel multiplexed approach to identify functional coregulators of chromatin effectors. ChECS enabled us to identify a so far unknown transcriptional regulatory cascade controlling developmental gene expression programs, which could contribute to oncogenesis in case of deregulation. Our study illustrates an interconnection of LSD1 and R-loop homeostasis, which provides novel insights into the biological functions of LSD1. Furthermore, we show for the first time that a DEAD-box helicase (DDX19A) contains a bona fide binding motif for selected histone modifications associated with gene repression and binds H3K27me3 with considerably high affinity (109). To date, DEAD-box helicases were not reported to interact with modified histone tails and do not harbour any of the so far known histone reading domains, a finding that might extend to additional RNA:DNA helicases, disclosing the potential for additional unknown regulatory pathways.

## DATA AVAILABILITY

LSD1 ChIP-seq data (GSE158441) is available via Gene Expression Omnibus (https://www.ncbi.nlm.nih.gov/geo/).

The following ChIP-seq tracks were obtained from published data sets in K562 cells: H3K27ac (Encode sample ENCFF384ZZM), H3K27me3 (Encode sample ENCFF936BVT), H3K9me3 (Encode sample ENCFF700FQH), H3K36me3 (Encode sample ENCFF223BKS), H3K4me1 (Encode sample ENCFF463AQS), H3K4me2 (Encode sample ENCFF778DNU), LSD1 (GEO sample GSM831002), R-ChIP (GEO sample GSM2551007/8), DRIP-seq (GEO sample GSM1720619), G4-ChIP-seq (GEO sample GSM2876090/1). K562 RNA-seq (GEO sample GSM1557077). The following ChIP-seq tracks were obtained from published data sets in NIH/3T3 cells: H3K9ac (GEO sample GSM1246687), H3K27me3 (GEO sample GSM1246690), H3K36me3 (GEO sample GSM1246692), H3K9me2 (GEO sample GSM1246688), H4ac (GEO sample GSM1418787), H3K4me3 (GEO sample GSM1246686), DRIPc-seq (GEO sample GSM2104456), DRIP-seq (GEO sample GSM1720621). NIH/3T3 RNA-seq (Encode sample ENCFF001QSC).

## Supplementary Material

gkab180_Supplemental_Files
