# Supplementary material for: A functional LSD1 coregulator screen reveals a novel transcriptional regulatory cascade connecting R-loop homeostasis with epigenetic regulation
Source: Nucleic Acids Res. 2021 Apr 6;49(8):4350–70. doi: 10.1093/nar/gkab180 (PMC8096265; doi:10.1093/nar/gkab180)
Supplement: gkab180_Supplemental_Files [file gkab180_supplemental_files.zip › Supplementary_information_V2_-_NAR-00447-X-2021.docx]

**A functional LSD1 coregulator screen reveals a novel transcriptional regulatory cascade connecting R-loop homeostasis with epigenetic regulation.**

Sabine Pinter^1^, Franziska Knodel^1^, Michel Choudalakis^1^, Philipp Schnee^1^, Carolin Kroll^1^, Marina Fuchs^1^, Alexander Broehm^1^, Sara Weirich^1^, Mareike Roth^3^, Stephan A. Eisler^2^, Johannes Zuber^3,4^, Albert Jeltsch^1^, Philipp Rathert^1*^

**Supplementary Material**

Supplementary Figure S1: The synP-mCherry reporter system is stably expressed in different cell lines and can be manipulated by external stimuli.

Supplementary Figure S2: Suppression of DDX19A expression interferes with LSD1 activity.

Supplementary Figure S3: DDX19A is involved in R-loop homeostasis and Ddx19a suppression leads to global accumulation of R-loops.

Supplementary Figure S4: Regions of LSD1 occupancy correlate with R-loops genome wide in K562 and NIH/3T3 cells.

Supplementary Figure S5: DRIP RNAseH1 controls with and without GSK-LSD1 treatment.

Supplementary Figure S6: DDX19A specifically binds trimethylated H3K27 and H4K20.

Supplementary Figure: Uncropped Western Blots.ai

Supplementary Tables S1-5.xlsx: Supplementary Table 1: ChECS primary data; Supplementary Table 2: ChECS gene scores; Supplementary Table 3: ChECS sequencing primer; Supplementary Table 4: shRNA guide sequences; Supplementary Table 5: qPCR primer sequences


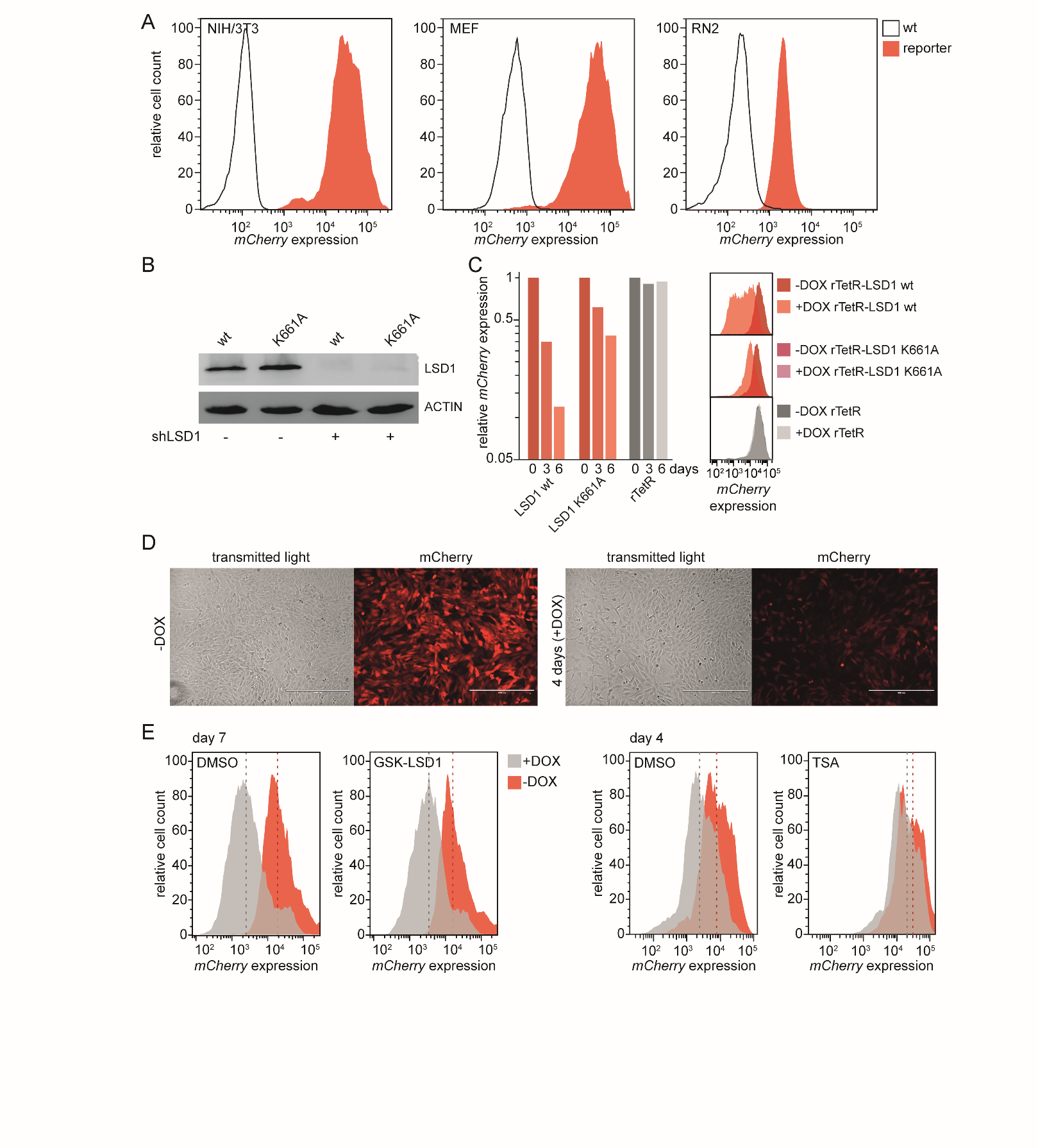


**Supplementary Figure S1: The *synP-mCherry* reporter system is stably expressed in different cell lines and can be manipulated by external stimuli. (A)** Flow-cytometric analysis of *mCherry* expression after transduction and selection of the *synP-mCherry* reporter cassette in different cell lines (red) compared to untransduced cells (grey). Signals were measured by flow cytometry and are normalized to the highest cell count. MEF=mouse embryonic fibroblasts; RN2=murine MLL–AF9;Nras^G12D^ AML cells. **(B)** Immunodetection of the expression levels of the rTetR-LSD1 wt or K661A with and without suppression by *shLSD1*. Whole-cell lysate of NIH/3T3 cells expressing the *synP-mCherry* reporter and the rTetR-LSD1 fusion protein was loaded. ACTIN was used as a loading control. **(C)** Flow cytometry analysis of NIH/3T3 during recruitment of rTetR-LSD1 wt, K661A or rTetR alone to the *synP* promoter. Bar graphs represent the median *mCherry* expression relative to the initial measurement. The histograms show *mCherry* expression profiles corresponding to the bar graphs at day 0 (-DOX) and day 6 (+DOX) of DOX treatment. One representative experiment is shown. **(D)** Representative fluorescence microscopy images of NIH/3T3 cells stably expressing the *synP-mCherry* reporter cassette together with rTetR-LSD1. Left: untreated cells (-DOX). Right: Cells with 4 days of LSD1 recruitment (+DOX). Scale bars are 400µm. **(E)** Representative *mCherry* expression profiles of NIH/3T3 cells after treatment with 10 µM GSK-LSD1, 400 nM TSA or DMSO and recruitment of rTetR-LSD1 by the addition of DOX for the indicated days. Grey: rTetR-LSD1 recruitment (+DOX), red: no recruitment (-DOX). Dotted lines represent the median *mCherry* signal of the respective population.


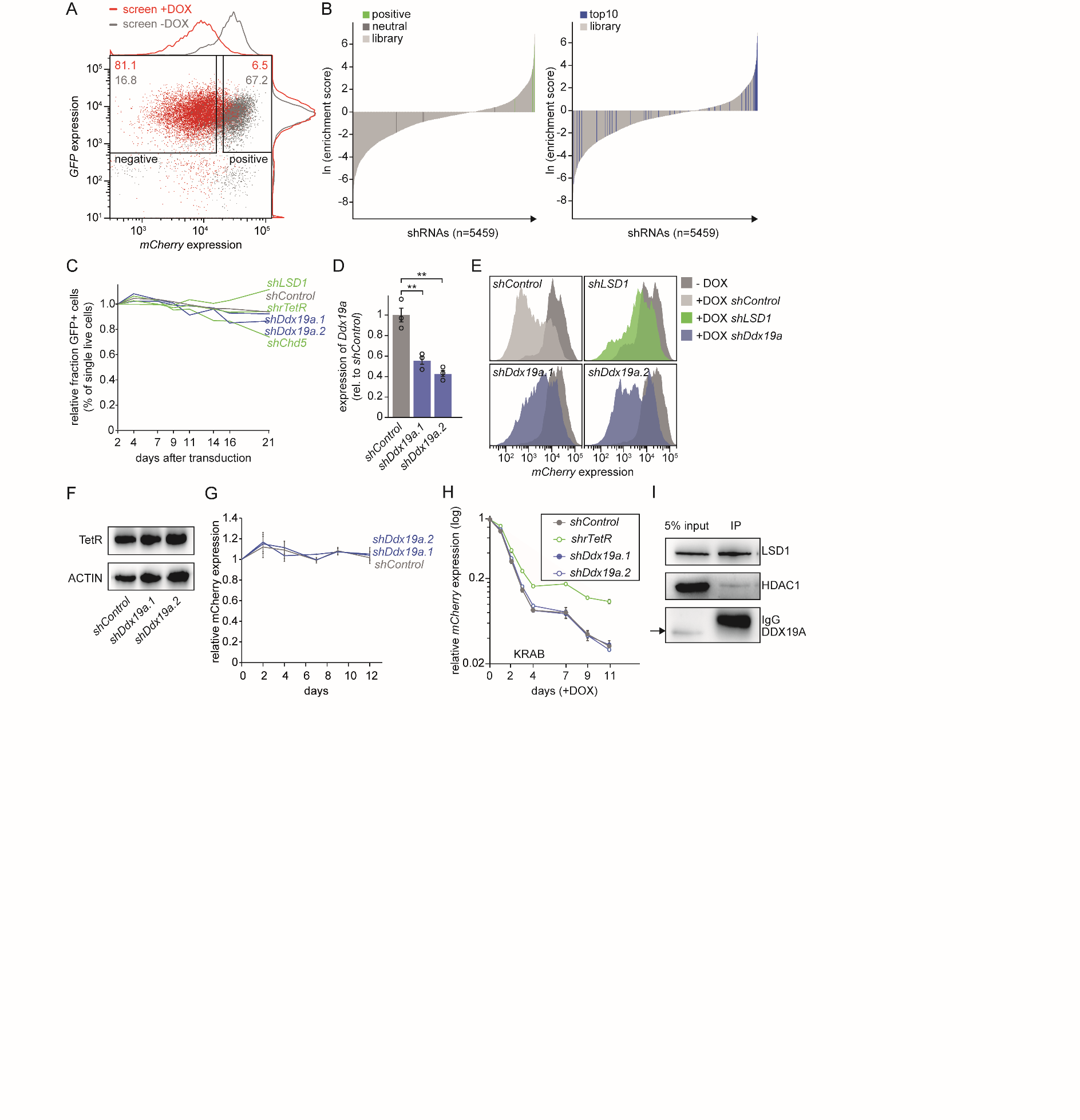


**Supplementary Figure S2: Suppression of DDX19A expression interferes with LSD1 activity. (A)** Scatter plot illustrating the gating strategy used in FACS. Red: representative cell population transduced with the shRNA library after 14 days +DOX. Grey: control population transduced with the shRNA library without the addition of DOX. Numbers indicate the percentage of cells that were sorted using the respective gate. Histograms of the respective populations are indicated. **(B)** Enrichment scores for all shRNAs tested in the screen. Enrichment scores for each shRNA were calculated by dividing the geometric mean of the read counts of all independent replicates (n=5) in the positive population by the geometric mean of the read counts of the negative population and are plotted as the natural logarithm (ln) in ascending order. Positive and neutral control shRNAs are highlighted in green and grey (left). shRNAs (4-6 per gene) targeting the top10 hits of the screen are highlighted in blue (right). **(C)** Competitive proliferation assays of NIH/3T3 cells expressing the indicated shRNAs. Shown is the fraction of GFP+/shRNA+ cells relative to the initial measurement. **(D)** qPCR analysis of *Ddx19a* mRNA levels in NIH/3T3 cells expressing *shDdx19a* or *shControl* for 10 days. Bars are relative to the *shControl* (n=3, mean±*s.e.m.*, p**≤0.01, *Student´s* t-test). **(E)** Validation of two shRNAs targeting *Ddx19a* (*shDdx19a.1* and *2*). Histograms showing representative *mCherry* expression profiles in rTetR-LSD1 reporter cell populations treated with DOX for 7 days. **(F)** Immunoblotting of NIH/3T3 cells expressing the rTetR-LSD1 fusion protein and the indicated shRNAs. Detection was performed with a TetR-specific antibody. ACTIN was used as a loading control. **(G)** Expression of the *mCherry* reporter in NIH/3T3 cells co-expressing the *synP-mCherry* reporter, the rTetR-LSD1 fusion protein under suppression of *Ddx19a* expression. Plotted is the median *mCherry* signal measured by flow-cytometry in cells expressing the indicated shRNAs without DOX treatment for the indicated number of days relative to the signal at day 0. The mean of individual replicates is plotted (n=3, mean±*s.e.m*.). **(H)** Time course of the *mCherry* signal in NIH/3T3 expressing the *synP-mCherry* reporter, a rTetR-KRAB fusion protein and the indicated shRNAs. Circles indicate the median *mCherry* expression under recruitment of rTetR-KRAB (+DOX) measured by flow cytometry relative to the initial measurement (n=3; mean±*s.e.m.*). **(I)** Immunoblotting of proteins co-precipitated with LSD1 from NIH/3T3 lysate using an antibody against LSD1. The strong band on the lowest blot originates from the antibody used for IP. The DDX19A band is highlighted with an arrow.


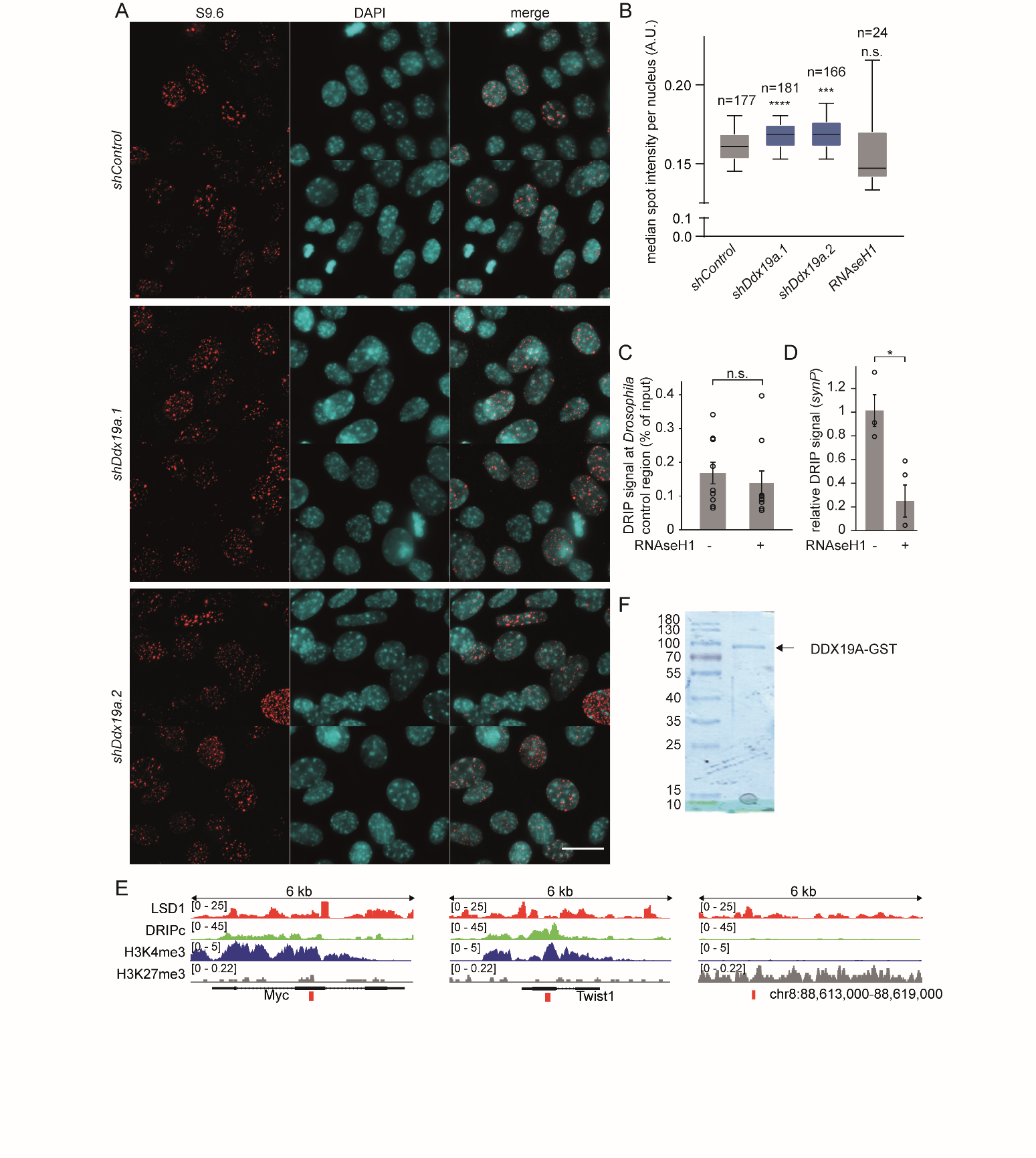


**Supplementary Figure S3:** **DDX19A is involved in R-loop homeostasis and Ddx19a suppression leads to global accumulation of R-loops. (A)** Representative immunofluorescence microscopy images of NIH/3T3 cells stably expressing the indicated shRNAs for 10 days and stained with the S9.6 antibody specific for R-loops as shown in Figure 3A. To capture more cells, a lower magnification than in Figure 3A is shown. Images are maximum intensity projections of a Z-stack covering the whole nucleus. Scale bar is 20 µm. **(B)** Box and whisker plot showing the analysis of the R-loop spot intensity per nucleus in NIH/3T3 cells from three independent experiments, stained with the S9.6 antibody as shown in (**A**). Images were analysed using CellProfiler™ software. Box-and-Whisker plots indicate the median and the 10-90 percentile (n=3; mean±*s.e.m.*, p***≤0.001, p****≤0.0001, n.s.=non-significant, *Student´s* t-test). [A.U.]=arbitrary unit. **(C)** Bar graph comparing the enrichment of spiked-in chromatin with the S9.6 antibody from samples that were treated with RNAseH1 before spike-in of *D.melanogaster* chromatin compared to the enrichment of spike-in *D.melanogaster* chromatin from samples that were not RNAseH1 treated indicating that the IP efficiency is comparable between RNAseH1 treated and untreated samples. (n=9; mean±*s.e.m.*, n.s.=not significant, Student´s *t*-test). Spike-in chromatin was not treated with RNAseH and used for the normalization of pulldown efficiency. **(D)** RNaseH1 control for the DRIP at the synP reporter element shown in Figure 3C. Nucleic acids were treated with RNAseH1 before performing the IP in parallel to the samples without RNAseH1 treatment. qPCR signals are shown relative to -RNAseH1 (n=3, p*<0.05, Student´s t-test). **(E)** Selected ChIP-seq occupancy profiles from NIH/3T3 cells at representative endogenous loci shown in Figure 3D. The regions amplified by qPCR after DRIP are indicated as red bars. **(F)** Coomassie-stained polyacrylamide gel after SDS-PAGE of DDX19A-GST purified with affinity chromatography. Numbers on the left indicate the size of the molecular weight marker in kDa. Arrow indicates the expected size of the full-length recombinant protein.


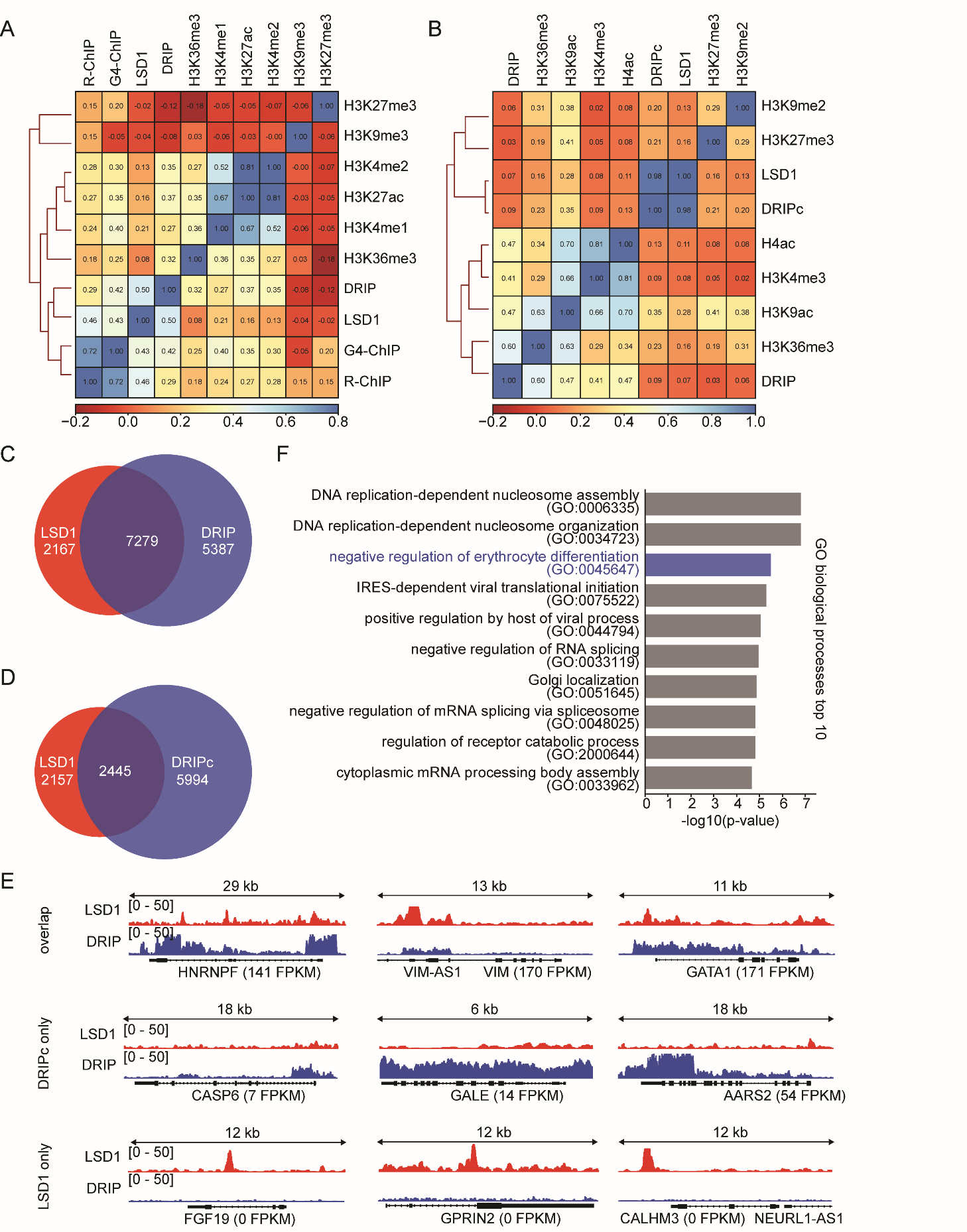


**Supplementary Figure S4: Regions of LSD1 occupancy correlate with R-loops genome wide in K562 and NIH/3T3 cells. (A), (B)** Correlation matrix of selected histone modifications and R-loop features in K562 (**A**) or NIH/3T3 cells (**B**) underlying the data shown in Figure 5C. The tracks were clustered according to their Pearson correlation coefficients. **(C)** and **(D),** Venn diagrams illustrating overlap of genes in the vicinity of LSD1 binding and/or R-loops in K562 (**C**) or NIH/3T3 cells (**D**). **(E)** Representative genomic regions in K562 cells showing the occupancy of LSD1 and R-loops at genes associated with the categories shown in Figure 4D. Relative expression is indicated for each gene as FPKM value. **(F)** Gene Ontology analysis of genes bound by LSD1 and R-loops. Genes were identified from LSD1 ChIP-seq and DRIPc-seq data from K562 cells and categorized for their annotated biological processes.

**
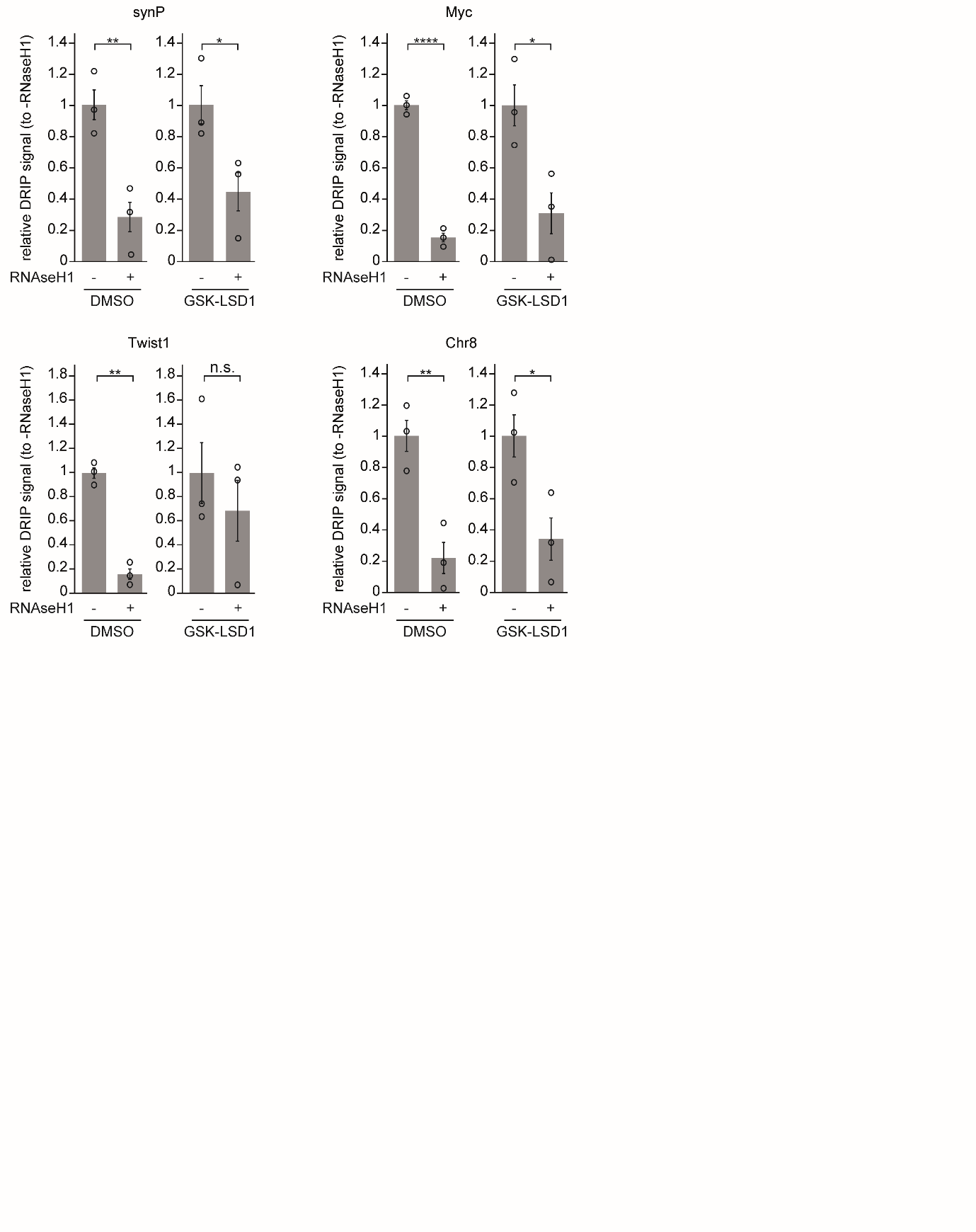
**

**Supplementary Figure S5: DRIP RNAseH1 controls with and without GSK-LSD1 treatment.** RNaseH1 controls for the DRIP analysis shown in Figure 5D at the indicated regions. Nucleic acids were treated with RNAseH1 before performing the IP in parallel to the samples without RNAseH1 treatment. qPCR signals are shown relative to -RNAseH1 (n=3, mean±*s.e.m.*, p*<0.05, p**<0.01, p****<0.0001, n.s.=not significant, Student´s t-test).


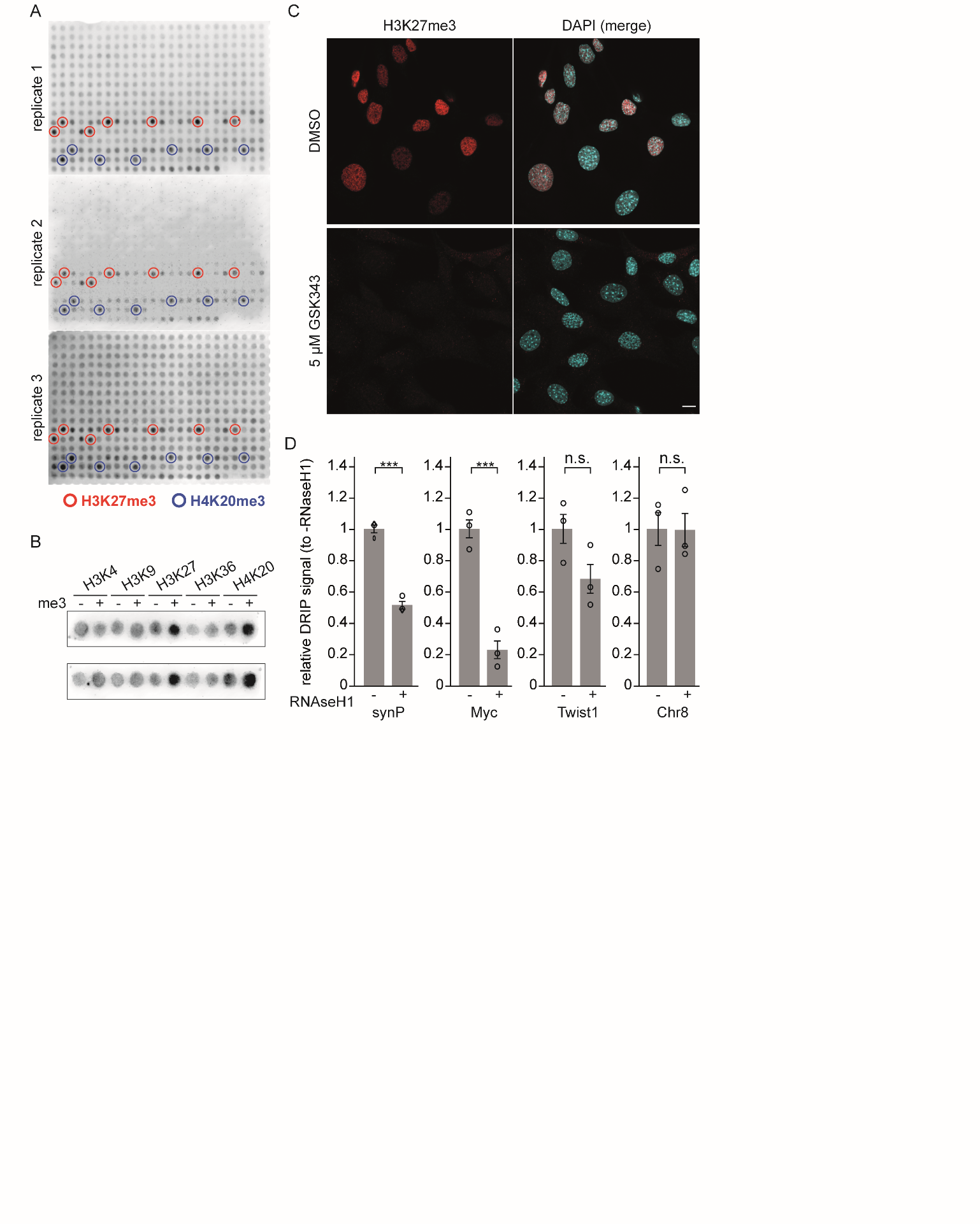


**Supplementary Figure S6: DDX19A specifically binds trimethylated H3K27 and H4K20. (A)** Independent replicates of the MODified™ Histone Peptide Arrays incubated with 50 nM DDX19A-GST. Binding was detected using an anti-GST antibody and an HRP-coupled secondary antibody. All peptides featuring H3K27me3 are highlighted in red, all peptides featuring H4K20me3 are highlighted in blue. The duplicates on the right side were left unlabelled for better visualization. **(B) Two** representative peptide SPOT arrays featuring the indicated unmodified and trimethylated histone peptides. Arrays were incubated with 50 nM DDX19A-GST. **(C)** Representative immunofluorescence images of NIH/3T3 cells treated with 5 µM GSK-343 or DMSO for 3 days and stained with an H3K27me3 specific antibody. Scale bar is 10 µm. DAPI was used as a nuclear marker. **(D)** RNaseH1 controls for DRIP at the indicated regions shown in Figure 6F. Nucleic acids were treated with RNAseH1 before performing the IP in parallel to the samples without RNAseH1 treatment. The qPCR signals are shown relative to -RNAseH1 (n=3, mean±*s.e.m.*, p***<0.001, n.s.=not significant, Student´s *t*-test).
