# Supplementary material for: A functional LSD1 coregulator screen reveals a novel transcriptional regulatory cascade connecting R-loop homeostasis with epigenetic regulation
Source: Nucleic Acids Res. 2021 Apr 6;49(8):4350–70. doi: 10.1093/nar/gkab180 (PMC8096265; doi:10.1093/nar/gkab180)

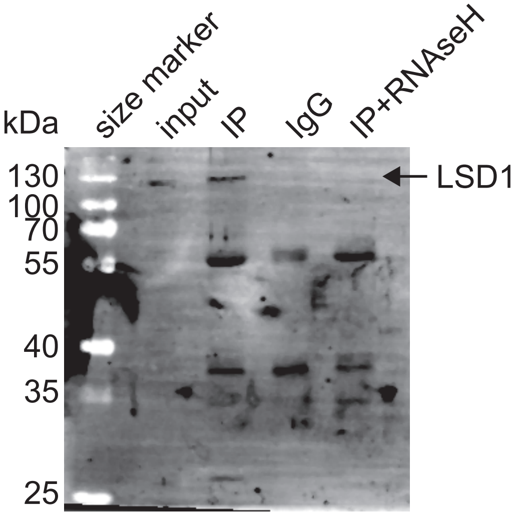

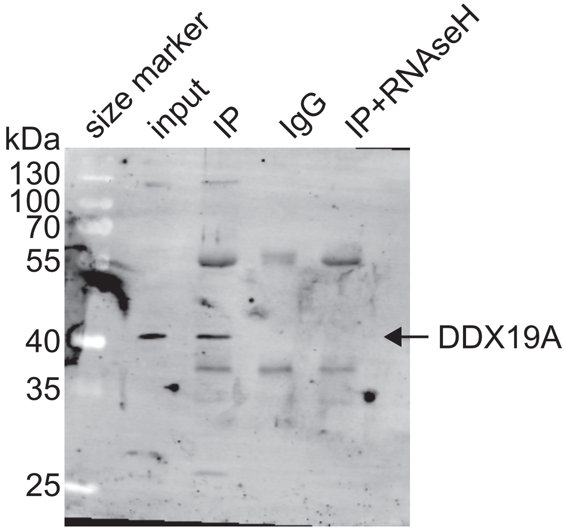

to **Figure 4E**: IP with the S9.6 antibody

---

to **Supplementary Figure 1b**: expression analysis of rTetR-LSD1 wt or K661A with and without suppression of LSD1 (*shLSD1)*.

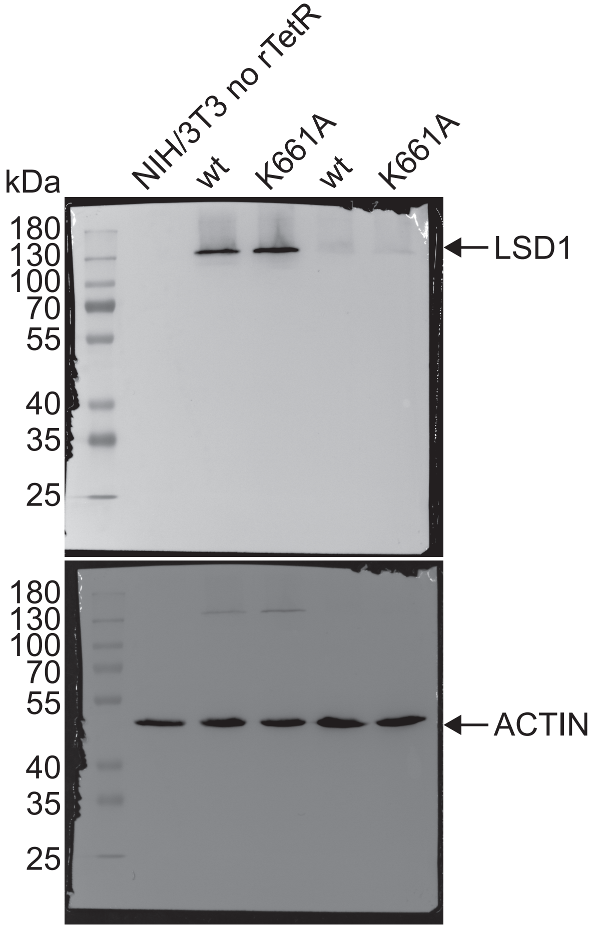

to **Supplementary Figure 2g:** IP with the LSD1 antibody.

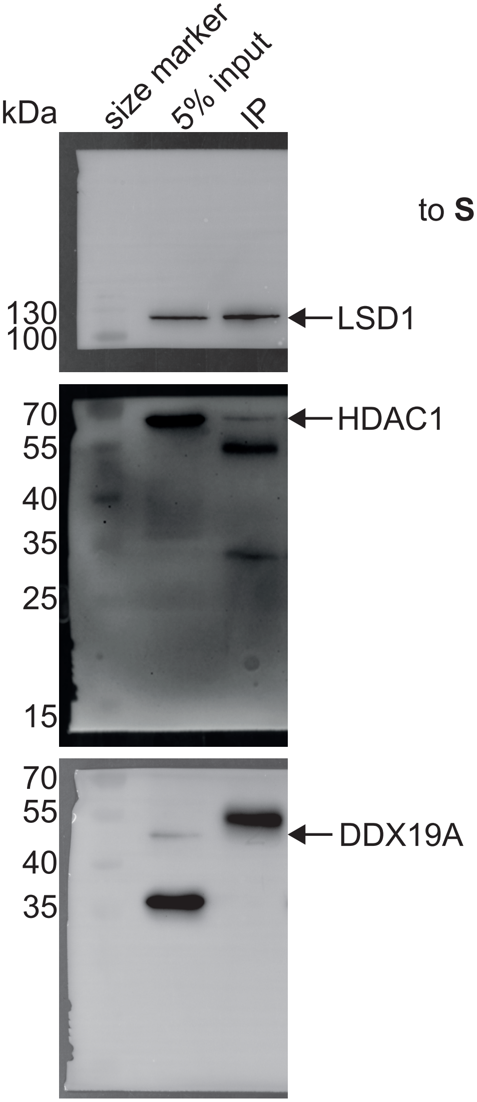

to **Supplementary Figure 2f:** expression of the TetR fusion protein under suppression of *Ddx19a*.

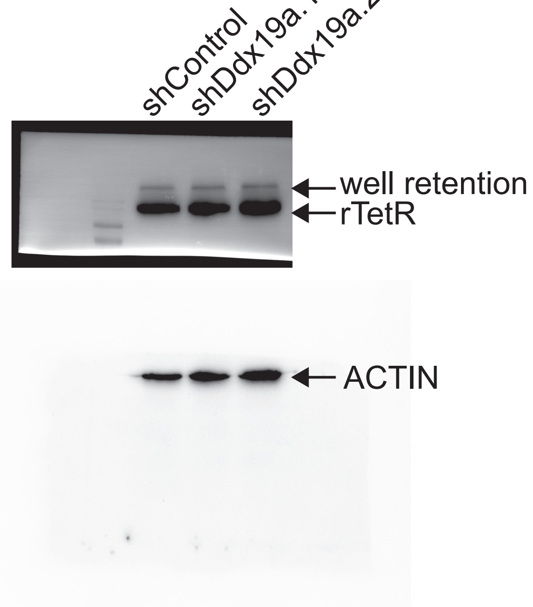

Supplement: gkab180_Supplemental_Files [file gkab180_supplemental_files.zip › Uncropped_Western_Blots.pdf]
